# Comprehensive profiling of the STE20 kinase family defines features essential for selective substrate targeting and signaling output

**DOI:** 10.1371/journal.pbio.2006540

**Published:** 2019-03-21

**Authors:** Chad J. Miller, Hua Jane Lou, Craig Simpson, Bert van de Kooij, Byung Hak Ha, Oriana S. Fisher, Natasha L. Pirman, Titus J. Boggon, Jesse Rinehart, Michael B. Yaffe, Rune Linding, Benjamin E. Turk

**Affiliations:** 1 Department of Pharmacology, Yale School of Medicine, New Haven, Connecticut, United States of America; 2 Biotech Research and Innovation Center, Faculty of Health and Medical Sciences, University of Copenhagen, Copenhagen, Denmark; 3 Departments of Biological Engineering and Biology, MIT Center for Precision Cancer Medicine and Koch Institute for Integrative Cancer Research, Massachusetts Institute of Technology, Cambridge, Massachusetts, United States of America; 4 Department of Cellular and Molecular Physiology and Systems Biology Institute, Yale University, New Haven, Connecticut, United States of America; Salk Institute for Biological Studies, United States of America

## Abstract

Specificity within protein kinase signaling cascades is determined by direct and indirect interactions between kinases and their substrates. While the impact of localization and recruitment on kinase–substrate targeting can be readily assessed, evaluating the relative importance of direct phosphorylation site interactions remains challenging. In this study, we examine the STE20 family of protein serine–threonine kinases to investigate basic mechanisms of substrate targeting. We used peptide arrays to define the phosphorylation site specificity for the majority of STE20 kinases and categorized them into four distinct groups. Using structure-guided mutagenesis, we identified key specificity-determining residues within the kinase catalytic cleft, including an unappreciated role for the kinase β3–αC loop region in controlling specificity. Exchanging key residues between the STE20 kinases p21-activated kinase 4 (PAK4) and Mammalian sterile 20 kinase 4 (MST4) largely interconverted their phosphorylation site preferences. In cells, a reprogrammed PAK4 mutant, engineered to recognize MST substrates, failed to phosphorylate PAK4 substrates or to mediate remodeling of the actin cytoskeleton. In contrast, this mutant could rescue signaling through the Hippo pathway in cells lacking multiple MST kinases. These observations formally demonstrate the importance of catalytic site specificity for directing protein kinase signal transduction pathways. Our findings further suggest that phosphorylation site specificity is both necessary and sufficient to mediate distinct signaling outputs of STE20 kinases and imply broad applicability to other kinase signaling systems.

## Author summary

Protein kinases, which catalyze the transfer of phosphate from ATP to substrate proteins, are important enzymes in cellular signal transduction pathways mediating responses to extracellular cues. In order to function properly in signal transmission, each kinase must phosphorylate only a limited number of proteins among the thousands present within the cell. One way that kinases choose their substrates is by recognition of amino-acid sequence motifs surrounding the site of phosphorylation, but kinases can also recruit substrates through docking interactions occurring outside of the catalytic cleft. For most kinases, the relative contribution of catalytic site and docking site interactions to substrate selection is not known. Here, we investigated the phosphorylation site specificity of a group of 30 human protein serine–threonine kinases called the STE20 family. Guided by x-ray crystal structures of kinase-peptide complexes, we re-engineered the catalytic clefts of two members of this group, PAK4 and MST4, to exchange their phosphorylation site motifs. In cells, the re-engineered form of PAK4 was unable to phosphorylate substrates of wild-type PAK4 or to carry out its normal function in reorganizing the actin cytoskeleton. In contrast, it was able to function in place of MST4 and related kinases in the growth-controlling Hippo signaling pathway. Overall, these studies suggest that catalytic site interactions can be both necessary and sufficient for kinase function.

## Introduction

Protein kinases are key enzymes in signal transduction networks critical to essentially all aspects of cellular regulation. The human genome encodes over 500 protein kinases that function in distinct processes yet share a structurally conserved catalytic domain [1]. The unique functions of each kinase are attributable to different modes of regulation (signal input) and different sets of protein substrates serving as their effectors (signal output). Classically, the capacity of kinases to target unique substrates was attributed to differences in the catalytic cleft that mediate recognition of distinct phosphorylation site consensus sequences [2]. However, even kinases belonging to the same family and sharing substantial sequence similarity within their catalytic domains can phosphorylate different substrate proteins [3]. There has been a growing appreciation that noncatalytic domains, adaptor proteins, and scaffolds can have important roles in substrate recruitment [4–6]. For closely related kinases, these interactions can override catalytic site specificity in driving substrate selection [7]. However, the extent to which phosphorylation site sequence motifs are necessary or sufficient to mediate selective signaling is not clear for most kinases. It is relatively straightforward to affect substrate recruitment through deletion or substitution of noncatalytic domains and binding partners. However, a lack of general approaches to perturb catalytic site interactions while maintaining kinase activity makes it difficult to assess their relative contribution to substrate targeting.

One prominent example of a closely related, yet functionally distinct, group of kinases is the STE20 family. The 30 serine–threonine kinases in this group have been classified into 10 subfamilies based on similarity within the catalytic domain and overall domain architecture (S1 Fig) [8]. Perhaps the best characterized are the two subfamilies of p21-activated kinases (PAKs), which function in growth factor and adhesion-dependent signaling pathways regulating the actin cytoskeleton to promote cell motility and survival [9]. More than 80 direct PAK substrates have been identified that collectively contribute to these functions [10, 11]. Most of the remaining STE20 kinases constitute multiple so-called germinal center kinase (GCK) subfamilies. Many GCKs transduce stress signals to promote cell cycle arrest and apoptosis, for example, by acting as upstream regulators of the c-Jun N-terminal kinase (JNK) and p38-mitogen–activated protein kinase (MAPK) cascades [12]. GCKs are also important upstream components of the tumor-suppressive Hippo pathway. Several of them, most prominently Mammalian sterile 20 kinase (MST)1 and MST2, phosphorylate the serine–threonine kinases large tumor suppressor homolog 1 and 2 (LATS1/2) within a C-terminal hydrophobic motif [13–16], promoting their activation and subsequent phosphorylation of the Yes-associated protein (YAP) and Transcriptional co-activator with PDZ binding motif (TAZ) transcription factors. Some GCKs, including thousand and one amino acid kinase 1 (TAO1) and TAO3, can also act further upstream through direct phosphorylation and activation of MST2 [17, 18].

Prior studies have largely emphasized the contribution of subcellular localization and noncatalytic site interactions to selective substrate targeting by STE20 kinases. For example, PAKs are localized and activated through interactions with small GTPases, producing an active pool of the kinase in a spatially restricted manner that likely limits its substrate repertoire. Multiple STE20 kinases, including some PAKs and GCKs, harbor proline-rich motifs that bind to Src homology 3 (SH3) domains present in substrates, activators, and/or adaptor proteins [19–22]. At least some members of the family (PAKs, TAOs, STE20/SPS1-related proline-alanine–rich kinase [SPAK]/Oxidative stress-responsive 1 [OSR1], and Lymphocyte-oriented kinase [LOK]) interact with defined regions of their substrates distal from sites of phosphorylation through either catalytic or noncatalytic domains [23–26]. Finally, several STE20 kinases have stable binding partners that may act as substrate adaptors [13, 14, 27–33].

Despite the established importance of noncatalytic site interactions, differences in phosphorylation site sequence motifs among STE20 kinases may also be functionally important. For example, the established catalytic site motif of PAKs corresponds well to sequences found at known sites of phosphorylation in protein substrates [34], albeit with some individual sites diverging substantially. Furthermore, subtle differences in the phosphorylation site sequence can direct different members of the PAK subfamily to unique substrates [35]. While peptide library analysis of a limited number of GCKs has revealed a specificity profile distinct from PAKs [36, 37], there has been no systematic analysis of the entire STE20 family, and specific determinants of specificity that distinguish members of the family are not known.

In this study, we probe the catalytic site specificity of the STE20 family and discover previously unknown features within the kinase domain that help determine selective substrate targeting. Exploiting these features, we engineer kinase mutants that fully exchange phosphorylation site specificity between subfamilies. These reprogrammed kinases provide an unprecedented opportunity to dissect the unique contribution of catalytic site interactions to the signaling output of a protein kinase. We show that phosphorylation site specificity has a predominant role in mediating substrate targeting in STE20-kinase–mediated signaling networks.

## Results

### Determination of STE20 family phosphorylation site motifs

To better understand how STE20 kinases mediate selective substrate targeting, we performed positional scanning peptide array (PSPA) screens across the entire family. We used a previously reported set of 198 peptide mixtures in which nine positions flanking a central phosphorylation site are systematically substituted with each of the 20 unmodified amino-acid residues, as well as phosphothreonine (pThr) and phosphotyrosine (pTyr) [38, 39]. In this method, we perform parallel radiolabel kinase assays in solution to determine relative rates of phosphorylation of each arrayed peptide mixture. We have previously reported PSPA analysis of five members of the PAK subfamily [35, 40, 41] and four additional kinases from other STE20 subfamilies [36, 42]. To enable efficient screening of the remaining STE20 family kinases, we modified the PSPA protocol [38, 39] to a semiautomated platform (see Materials and methods). Optimization of this protocol improved interassay reproducibility over the fully manual assay format (S2 Fig), consumed less kinase, and allowed more efficient multiplexing.

**Fig 1:**
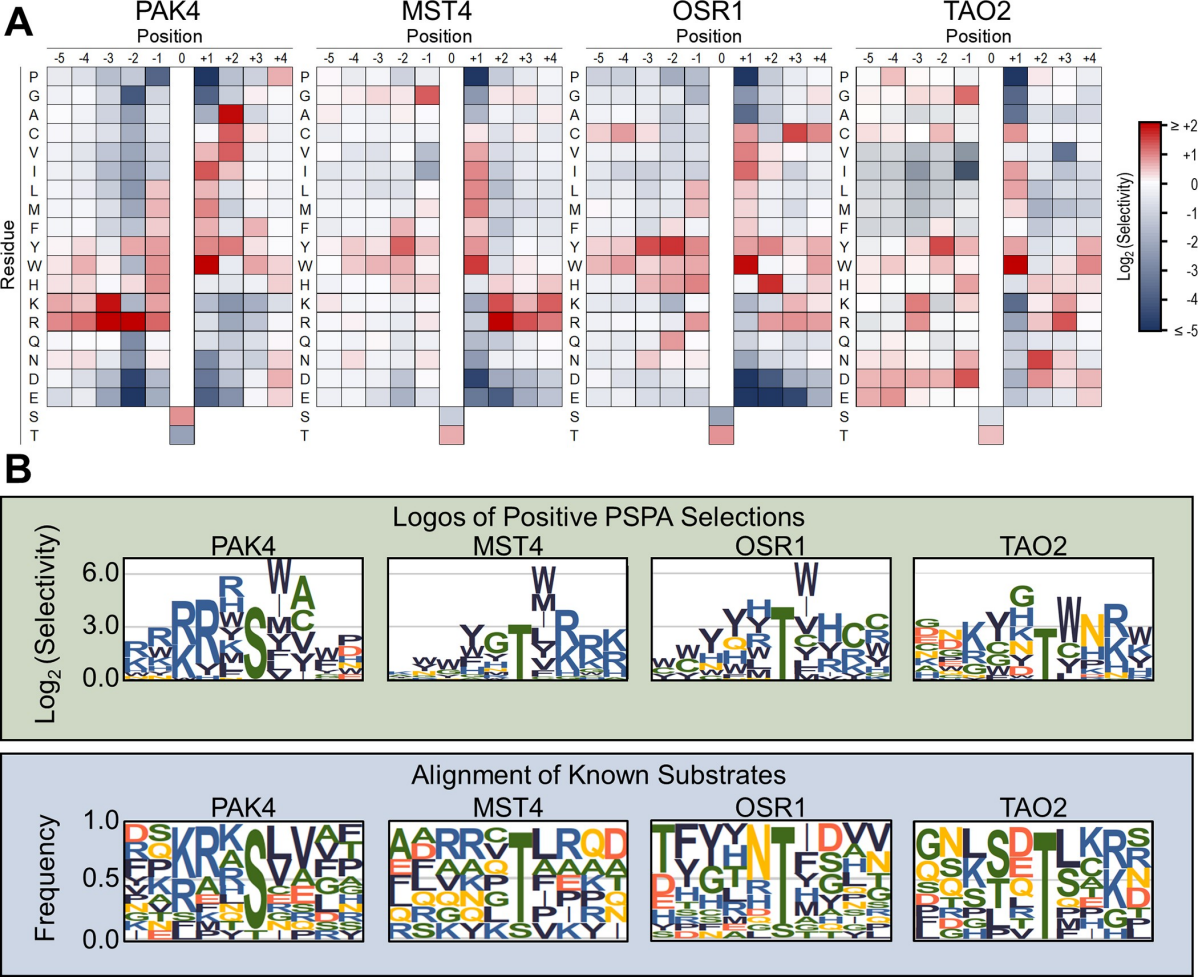
PSPA analysis showing representatives of the four major STE20 specificity groups. (A) Quantified PSPA spot intensities were normalized to an average value of 1 at each position within the peptide. Log_2_ transformed data are depicted as heat maps showing positively and negatively selected residues by position. Data are the mean of at least two separate experiments. Numerical values are provided in S1 Data. (B) Top: Positively selected residues from PSPA analysis for the same kinases as in panel A are shown as sequence logos. Bottom: Sequence logos based on alignments of all known phosphorylation sites mapped on protein substrates for the indicated kinase. Substrate phosphorylation sites were collected from the PhosphoSitePlus database, with the exception of TAO2 substrate sites that were identified in [43]. Logos were generated with enoLOGOS [44]. MST, Mammalian sterile 20 kinase; OSR1, Oxidative stress-responsive 1; PAK, p21-activated kinase; PSPA, positional scanning peptide array; TAO2, thousand and one amino acid kinase 2

Using the semiautomated PSPA protocol, we profiled most of the STE20 kinases not previously analyzed. Together with previously published results, we have collected PSPA data in total for 20 of the 28 active STE20 kinases (data for representative kinases are shown in Fig 1, with remaining kinases in S3 Fig; quantified data for all PSPA experiments are provided in S1 Data). The remaining eight kinases were either unavailable for screening or provided insufficient signal above background in the PSPA; two members of the family (STE20-related kinase adapter protein [STRAD]α and STRADβ) are pseudokinases and were not analyzed. With the exception of the myosin-III (MYO3) kinases (GCK-VII subfamily), our data include at least one member of each subfamily within the STE20 group (S1 Fig). Because available PSPA data for closely related members of the same subfamily were indistinguishable from each other, it is likely that the remaining kinases (SPAK, Kinase homologous to SPS1/STE20 1 [KHS1], NIK-related protein kinase [NRK], TAO1, TAO3, and PAK3) have profiles similar if not identical to their closest relatives. For nine kinases, we also produced and assayed a kinase-inactive mutant form. We observed no detectable activity with any of these kinase-inactive mutants, suggesting that our results with the wild-type (WT) kinases were not attributable to contaminants in our purified preparations.

Based on the PSPA screens, we could categorize the family into four groups based on their target motifs (Fig 1A). The only common feature consistently recognized by all STE20 family kinases was a hydrophobic residue immediately downstream of the phosphorylation site (the +1 position). The PAKs were notably divergent from all of the other kinases we screened, having, as previously reported, a primary preference for basic amino acids at the −2 and −3 positions and for Ser over Thr as the phosphoacceptor residue. By contrast, all other members of the STE20 family shared a strong preference for basic residues at multiple positions downstream of the phosphorylation site, selected Thr over Ser as the phosphoacceptor, and preferred an aromatic residue at the −2 position. Kinases outside of the PAK subfamily could be further subdivided into three categories based on variations within this core motif or by differences observed at other positions. The largest category, comprising 11 of the kinases we screened across four subfamilies (hereafter referred to as the GCKi–v group), strongly preferred Lys or Arg residues clustered at the +2 and +3 positions (Fig 1, S3 Fig, and S4 Fig). By contrast, the kinase OSR1 preferred a His residue at the +2 position as well as the −1 position and showed a preference for a Tyr residue at the −3 position. TAO2 preferred Asn at the +2 position and also selected phosphorylated amino acids (pThr and pTyr) at several positions, suggesting that its substrates may be “primed” by prior phosphorylation at nearby sites (Fig 1 and S1 Data). These categories aligned well with the phylogenetic relationships within the STE20 group (S1 Fig and S4 Fig).

The residues most strongly selected by most STE20 kinases as determined by PSPA were over-represented in previously reported phosphorylation sites in protein substrates (Fig 1 and S3 Fig) with some exceptions. For example, while Trp was selected most strongly at the +1 position by all STE20 kinases, authentic protein substrates reflected the general preference for hydrophobic residues at this position seen by PSPA analysis. Furthermore, basic residues were less common at positions downstream of the phosphorylation site in MST4 (and MST1/2; S3 Fig) protein substrates than would be expected from their strong enhancement of peptide substrate phosphorylation. This observation could reflect redundancy in which the presence of basic residues at all three downstream positions is not required for maximal activity.

**Fig 2:**
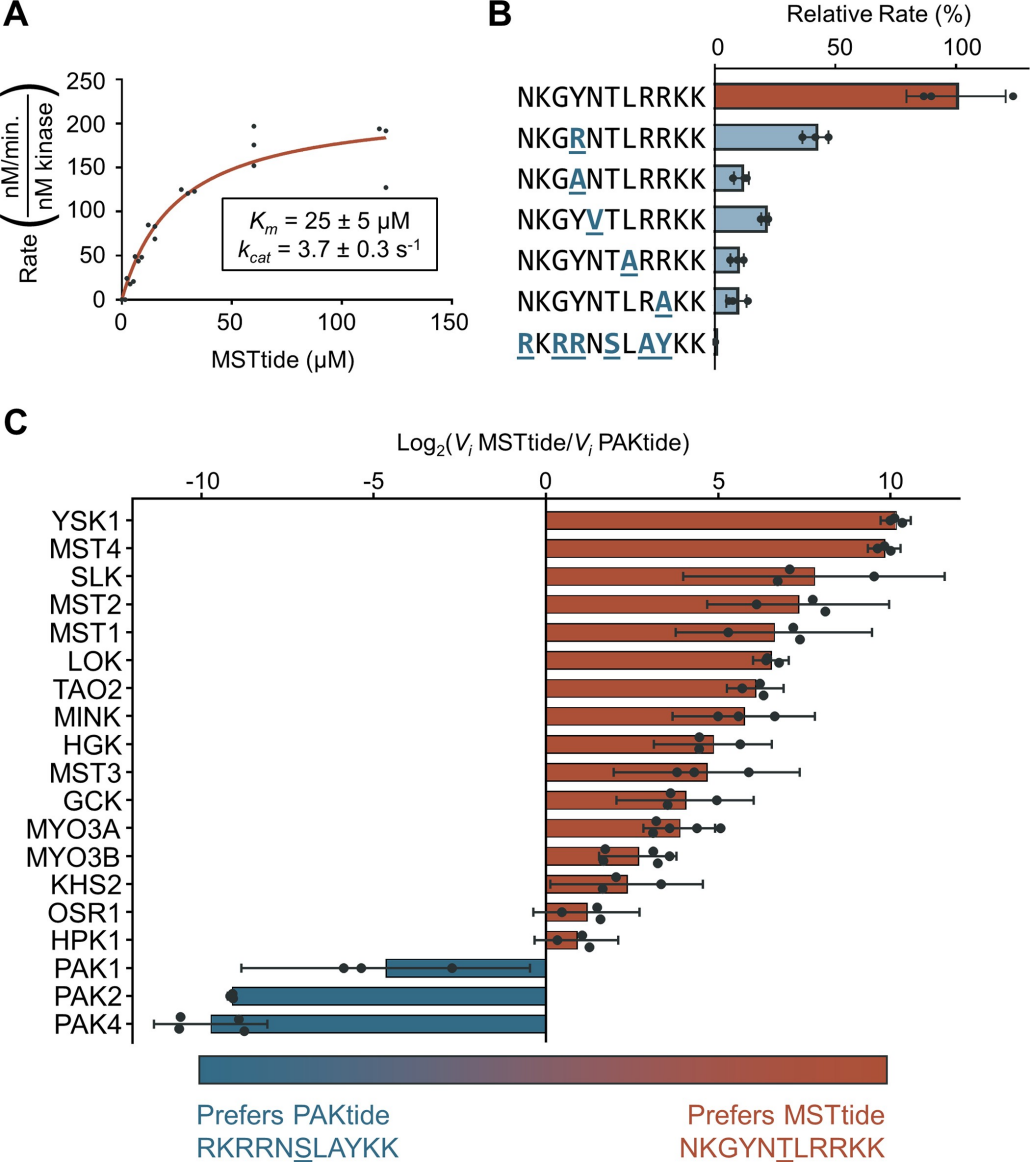
Phosphorylation kinetics of peptide substrates by STE20 kinases. (A) Michaelis–Menten curve for MST4 phosphorylation of MSTtide. Individual data points from three separate experiments are shown. (B) Initial rates of phosphorylation by MST4 of a series of peptides with the indicated sequences (*n* ≥ 3, bars show mean ± SD) shown relative to MSTtide (top) phosphorylation. Data for PAKtide are at bottom. (C) Relative initial rates of phosphorylation of MSTtide and PAKtide by a series of STE20 kinases (*n* = 3, error bars are 95% CI). Kinases with bars that do not cross the *y*-axis have a statistically significant preference for one consensus peptide over the other (*p* = 0.05). Source data for all panels are provided in S2 Data. GCK, germinal center kinase; HGK, HPK/GCK-like kinase; HPK1, Hematopoietic progenitor kinase 1; KHS, Kinase homologous to SPS1/STE20; LOK, Lymphocyte-oriented kinase; MINK, Misshapen-like kinase 1; MST, Mammalian sterile 20 kinase; MYO, myosin; OSR1, Oxidative stress-responsive 1; PAK, p21-activated kinase; SLK, STE20-like kinase; TAO, thousand and one amino acid kinase; YSK1, Yeast Sps1/Ste20-related Kinase 1

While the phosphorylation site motif of PAKs has been explored in depth [35, 45], there has been comparatively little characterization of other STE20 kinases. To more quantitatively assess the contribution of specific residues to phosphorylation by kinases outside the PAK subfamilies, we designed a peptide substrate (MSTtide, NKGYNTLRRKK) incorporating residues selected by these kinases but specifically optimized for the GCKi–v group that includes MST4 (S3 Fig). As anticipated, MST4 phosphorylated this peptide robustly (*K*_*m*_ = 25 ± 5 μM, *k*_*cat*_ = 3.7 ± 0.3 s^−1^, Fig 2A) . Amino-acid substitutions at several positions within the peptide reduced the phosphorylation rate between 2- and 10-fold, with the most substantial decreases observed with substitutions at the +1 and +3 positions (Fig 2B). We previously reported a similar reduction in MST4 peptide kinase activity upon exchanging a Ser residue for a Thr phosphoacceptor [46]. MST4 did not detectably phosphorylate a consensus PAK peptide substrate (PAKtide, RKRRNSLAYKK), indicating that combined substitutions at multiple positions further decrease the phosphorylation rate. To confirm trends in specificity observed across the entire STE20 family, we compared the phosphorylation rate of MSTtide to PAKtide for an additional 19 kinases, including the two MYO3 kinases that did not provide a robust PSPA signal. While PAKs detectably phosphorylated only PAKtide, all other kinases preferred MSTtide over PAKtide, albeit to varying degrees (Fig 2C). Overall, these assays confirm the importance of key substrate residues selected by multiple STE20 kinase subfamilies.

### Determinants of STE20 family kinase phosphorylation site specificity

**Fig 3:**
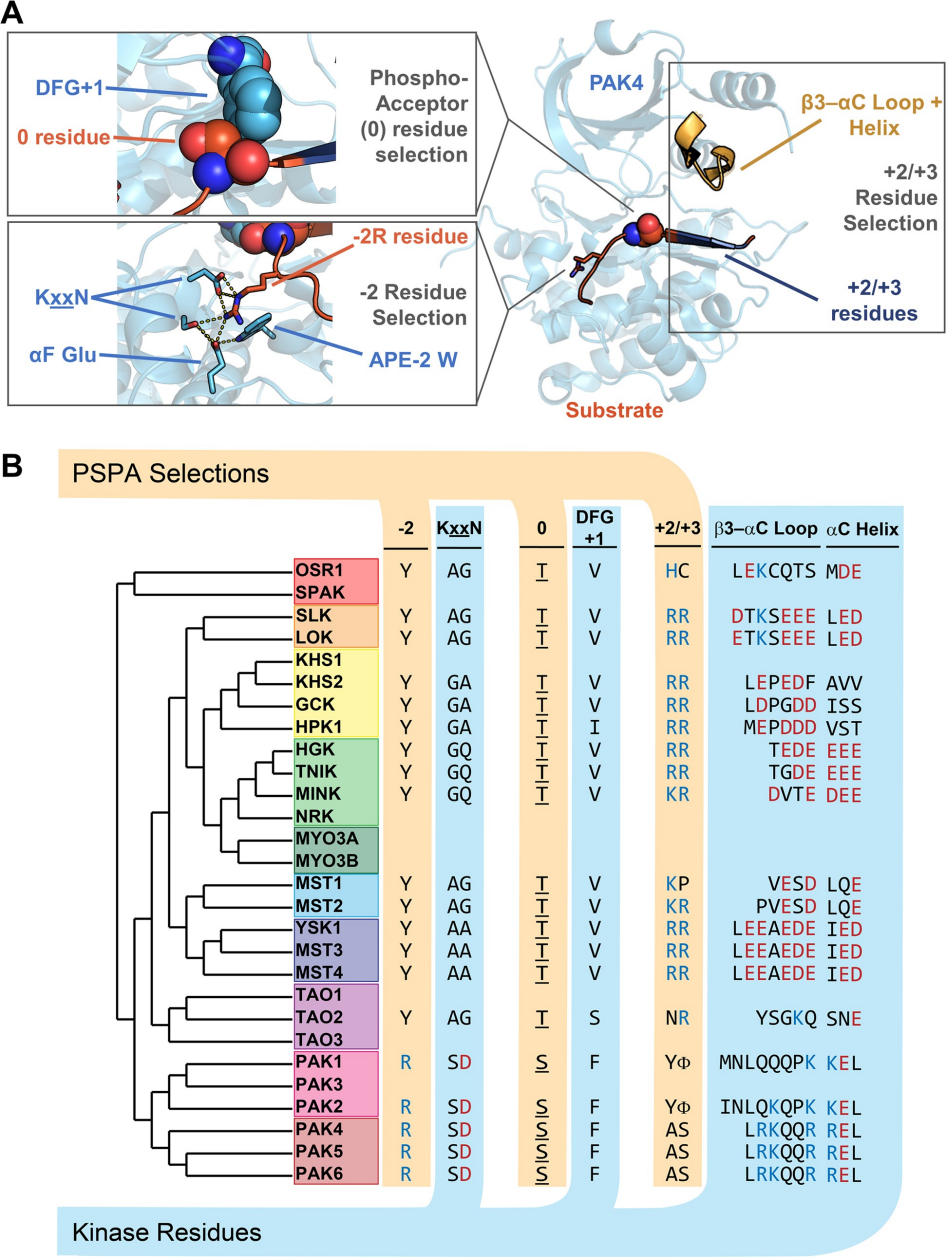
Overview of putative specificity-determining residues in the STE kinase family. (A) Regions of the kinase proximal to the −2 (KxxN motif), 0 (DFG+1), and +2/+3 (β3–αC loop region) residues are highlighted on the x-ray crystal structure of PAK4 in complex with a peptide substrate (PDB: 2Q0N). (B) Dendrogram of the STE20 kinase family showing putative specificity-determining regions and corresponding residues selected by PSPA analysis. The alignment of the β3–αC loop region was made using data from available x-ray crystal structures and predictions from PSIPRED v3.3 [49, 50]. GCK, germinal center kinase; HGK, HPK/GCK-like kinase; HPK, Hematopoietic progenitor kinase 1; KHS, Kinase homologous to SPS1/STE20; LOK, Lymphocyte-oriented kinase; MINK, Misshapen-like kinase 1; MST, Mammalian sterile 20 kinase; MYO, myosin; NRK, NIK-related protein kinase; OSR1, Oxidative stress-responsive 1; PAK, p21-activated kinase; PDB, Protein Data Bank; PSPA, positional scanning peptide array; SLK, STE20-like kinase; SPAK, STE20/SPS1-related proline-alanine–rich kinase; TAO, thousand and one amino acid; TNIK, Traf2 and NCK-interacting protein kinase; YSK1, Yeast Sps1/Ste20-related Kinase 1

**Fig 4:**
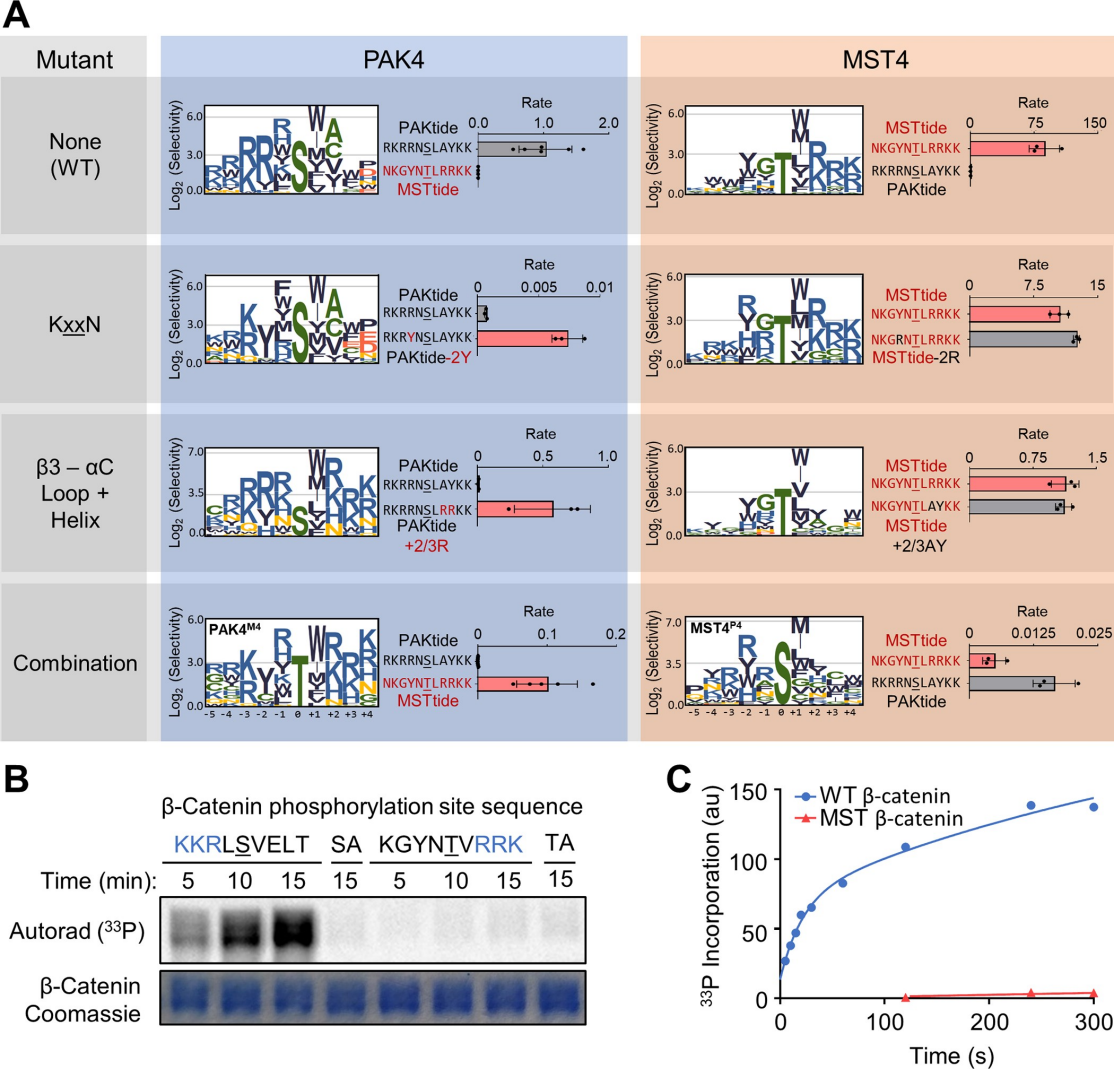
Mutation of specificity-determining residues exchanges substrate specificity between PAK4 and MST4. Mutants harbor residues found at the indicated positions in the other kinase as described in the main text. The combination mutants (PAK4^M4^ and MST4^P4^) include KxxN, DFG+1 and β3–αC loop region mutants (and S445N for PAK4^M4^). (A) Logos show positively selected residues from PSPA analysis of the indicated mutant kinases. Data were processed and visualized as in Fig 1. Bar graphs show peptide kinase assays with the indicated pairs of peptide substrates (*n* = 3, error bars indicate SD, rate units are nM/min/nM kinase). (B) Autoradiograph shows in vitro phosphorylation of full-length β-catenin variants with the indicated sequences surrounding Ser675 by WT PAK4. SA and TA are the corresponding S675A or T675A mutants. All β-catenin constructs included two additional mutations to remove minor sites of phosphorylation (S552A/T556A). (C) The graph shows the level of WT PAK4 phosphorylation of β-catenin variants used in (B) over time under pre-steady–state conditions. Numerical data for (A) and (C) are in S2 Data. MST, Mammalian sterile 20 kinase; PAK, p21-activated kinase; PSPA, positional scanning peptide array; WT, wild type.

Kinases target specific phosphorylation site sequences through complementary interactions within the catalytic cleft. While some insight into the structural basis for kinase–substrate recognition has been obtained from crystallographic studies and site-directed mutagenesis, only a few bona fide specificity-determining residues have been experimentally validated [45–48]. To identify specific kinase residues responsible for our observed substrate selectivity, we analyzed the x-ray crystal structures of PAK4-peptide complexes [46]. In the crystal structures, the guanidino headgroup of the −2 Arg residue in the peptide occupies an acidic pocket comprising two acidic residues (Asp444 and Glu507) and a polar Ser residue (Ser443) (Fig 3A). This pocket has previously been implicated in mediating specificity at the −2 position for PAKs and other kinases, including cAMP-dependent protein kinase (PKA) [45]. Glu507 is found in helix αF of the kinase domain, and an acidic residue is found at the analogous position in all STE20 kinases. All STE20 kinases also have a Trp residue (Trp481 in PAK4) located two residues upstream of the conserved Asp-Pro-Glu motif within the kinase activation loop (the APE-2 position) that orients Glu507 through a direct hydrogen bonding interaction (Fig 3A). Thus, while these residues are likely required for selection of Arg at the −2 position, they cannot function to determine specificity within this group. The other two residues in this pocket, Ser443 and Asp444, are located within a conserved KxxN sequence in the kinase catalytic loop. Almost all STE20 family kinases other than the PAKs have small residues, Gly or Ala, at both positions, potentially creating a cavity that could accommodate the larger aromatic residues observed by PSPA analysis (Fig 3B). Furthermore, these residues are highly conserved across animals, fungi, and protists: the KxxN residues of the closest PAK4 homologs from representative species were invariably Ser–Asp and were either Ala–Ala or Ala–Ser in all MST4 orthologs (S5 Fig). To assess their importance in determining specificity, we generated PAK4 and MST4 mutants in which the KxxN residues were exchanged between the two kinases. We conducted both PSPA analysis and assays with a −2 Tyr substituted PAKtide. In both settings, PAK4^S443A/D444A^ lost its strong preference for Arg and subsequently selected Tyr at the −2 position (Fig 4A). Similarly, the corresponding MST4 mutant (MST4^A147S/A148D^) displayed increased activity on peptides with a −2 Arg residue. We note that mutation of the −2 interaction pocket of both kinases was accompanied by a decrease in overall catalytic rate. This effect was particularly pronounced for PAK4, resulting in an approximately 1,000-fold decrease in the rate of phosphorylation of the parental PAKtide. While PAK4^S443A/D444A^ did phosphorylate the PAKtide −2Y variant faster than did WT PAK4, a large component of the change in selectivity appears to be conferred by loss of activity on the parental peptide. Overall, these assays confirm that the KxxN residues largely confer specificity at the −2 position in STE20 kinases.

A common feature of the GCKi–v group is strong selectivity for basic residues at multiple positions downstream of the phosphorylation site. We noted that in the PAK4–peptide structure, these residues were situated proximal to the loop connecting the β3 strand and the αC helix in the kinase N-lobe (Fig 3A). Among STE20 kinases, the net charge of this loop and the N-terminal region of helix αC correlated with selectivity at positions downstream of the phosphorylation site as seen by PSPA analysis (Fig 3B). For example, in GCKi–v kinases, this region is rich in acidic residues, suggesting complementary interactions with basic residues in the substrate. Interestingly, OSR1 and TAO2, which select basic residues at only one position, tend to have fewer acidic residues that are balanced by some basic residues in this region (Fig 3B). Finally, PAKs carry a net positive charge in this region, consistent with their strong selection against basic residues at these positions. These trends are also evolutionarily conserved because all PAK orthologs examined had a net positive charge in this region, while the closest MST4 homologs each had between five and seven acidic residues (S5 Fig). We hypothesized that attractive or repulsive electrostatic interactions serve to drive substrate specificity at the +2 to +4 positions. Exchanging the MST4 β3–αC loop with that of PAK4 (MST4^58–66→RKQQRREL^) led to loss of selectivity for basic residues at these positions in the PSPA and caused a 70-fold decrease in the rate of phosphorylation of MSTtide. Furthermore, the activity of this mutant was not impaired by neutralizing the +2 and +3 basic residues of MSTtide (Fig 4A). The corresponding PAK4 loop exchange mutant, PAK4^355–362→EEAEDEIED^, gained strong preferences for basic residues at positions +2 to +4 (Fig 4A). This mutant no longer detectably phosphorylated PAKtide, but substituting its +2 and +3 residues with Arg recovered activity to within 2-fold of the WT kinase on the parental peptide. These results suggest that basic residues at positions downstream of the phosphoacceptor greatly enhance activity of STE20 kinases and establish the β3–αC loop region as a strong determinant of substrate specificity within the STE20 kinase family.

The above analyses of PAK4 specificity were performed on peptide substrates, which cannot recapitulate noncatalytic site interactions found with full-length protein substrates. Furthermore, these experiments were performed under steady-state conditions that may not simulate a cellular context in which kinases may be present in excess of their substrates and in which phosphorylation often progresses to full stoichiometry. To extend these results to a true protein substrate, we performed kinase assays using the established PAK4 substrate β-catenin. The major PAK4 phosphorylation site on β-catenin, Ser675, is found in a sequence context (KKRL**S**VELT) that conforms well to our defined PAK4 consensus motif, including basic residues at the −3 and −2 positions. To assess the importance of PAK4 catalytic site interactions, we mutated this phosphorylation site to a sequence preferred by GCKi–v kinases (KGYN**T**VRRK). Similar to our analysis of peptide substrates, in steady-state kinetic assays, we found that this mutation reduced β-catenin phosphorylation by PAK4 to background levels (Fig 4B). To examine phosphorylation under non-steady–state conditions, we performed short time-course experiments at high PAK4 concentration to capture single turnover events. Under these conditions, we observed burst kinetics in which the first turnover occurred faster than the steady-state rate (Fig 4C). This observation suggests that product release is at least partially rate limiting for PAK4 phosphorylation of β-catenin. Importantly, we found that mutation of the sequence surrounding the phosphorylation site dramatically decreased the single turnover rate in addition to the steady-state rate. We conclude that catalytic site interactions accelerate a step in the kinase reaction prior to product release, either substrate association or phosphate transfer. Consequently, in the presence of a nonoptimal phosphorylation site, product release is no longer the rate-limiting step. While the sequence surrounding Ser675 appears to be nearly optimal for PAK4, PSPA analysis suggested that changing the residue at the +1 position to a bulkier Trp residue would increase the rate of phosphorylation. Contrary to anticipation, we found PAK4 to phosphorylate the V676W mutant approximately 2-fold more slowly than WT β-catenin (S6A Fig). This observation could mean that the presence of a bulky hydrophobic residue near the phosphorylation site may hinder substrate dissociation. However, we found in kinetic burst experiments that the rate of the first turnover was also decreased with this mutant (S6B Fig). It may be that the optimal residue at the +1 position is context-dependent, such that the β-catenin sequence confers a preference for Val rather than Trp.

**Fig 5:**
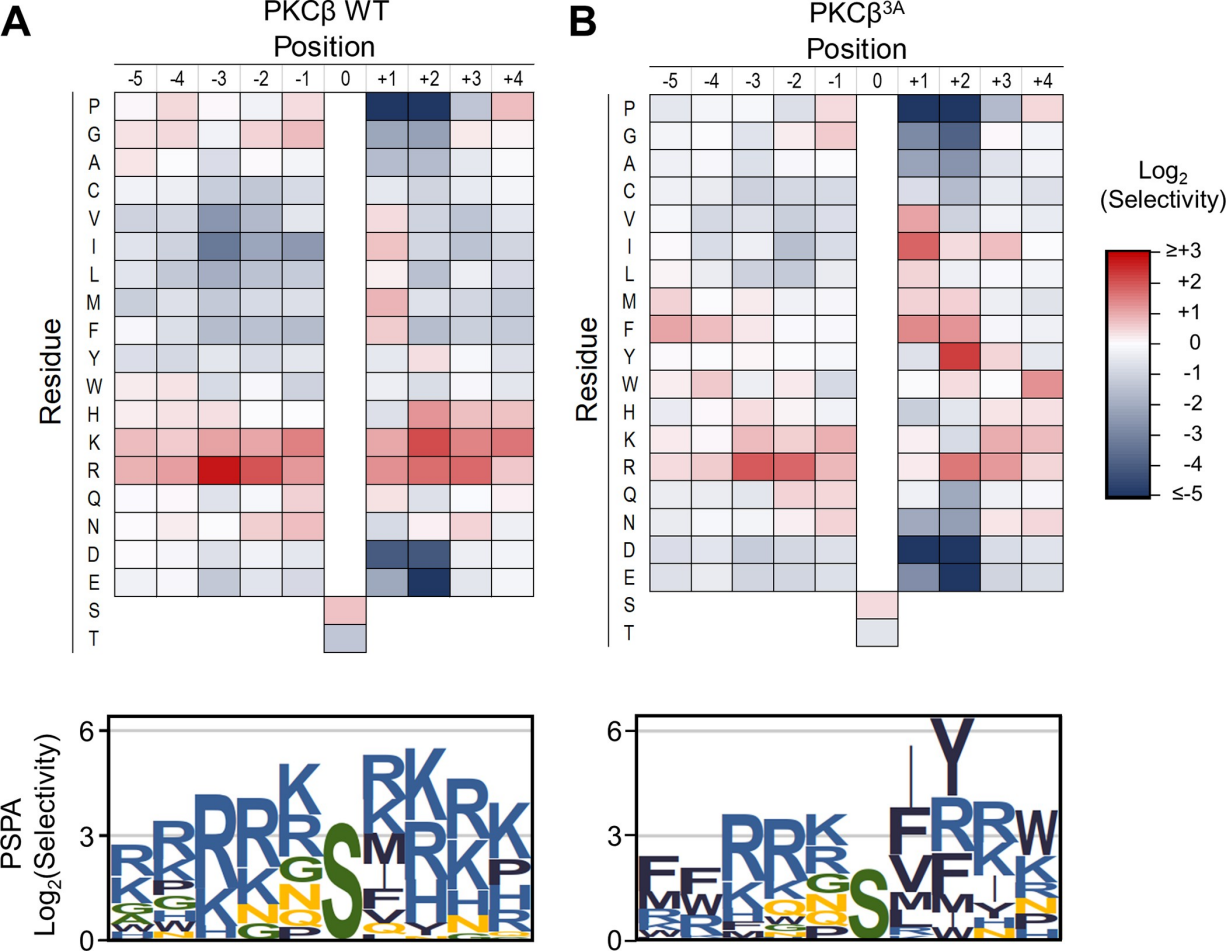
Acidic residues in the β3–αC loop region promote selection of basic residues by PKCβ. PSPA analysis shown as heat maps and sequence logos (prepared as in Fig 1) for WT (A) and the β3–αC loop mutant (B) of PKCβ. Numerical data are provided in S1 Data. PKC, protein kinase C; PSPA, positional scanning peptide array; WT, wild type.

Like GCKi–v kinases, all members of the protein kinase C (PKC) family select basic residues downstream of the phosphorylation site, primarily at the +2 position. Though PKC isozymes have low sequence similarity to STE20 kinases, their β3–αC loops are also rich in acidic residues. To determine whether this region might also confer selectivity for basic residues to PKCs, we examined a PKCβ mutant in which three acidic residues in the loop were mutated to Ala (PKCβ^3A^). PSPA analysis showed that this mutant had significantly reduced basic preferences at multiple positions and preferred aromatic hydrophobic residues at the +2 position (Fig 5). We conclude that the β3–αC loop region can act generally as a determinant of specificity among disparate kinase groups.

### Catalytic site interactions are important for signaling downstream of PAK4

Reprogrammed kinase mutants can provide tools to assess how particular elements of substrate specificity contribute to their signaling output. To fully re-engineer the catalytic site specificity of MST4 and PAK4, we combined the KxxN and β3–αC loop mutations and also exchanged a residue previously shown to mediate Ser versus Thr phosphoacceptor specificity (the residue immediately downstream of the conserved Asp-Phe-Gly sequence in the activation loop, termed the DFG+1 residue) [46]. Enzyme engineering frequently results in loss of catalytic activity, and we found that both exchange mutants had substantially reduced activity compared to their WT counterparts as assessed on their favored peptide substrates. To improve the activity of the PAK4 mutant, we added an additional mutation reported to increase PAK4 activity (S445N) [51]. The resulting compound mutants (termed PAK4^M4^ and MST^P4^) largely exchanged the substrate specificity of the two kinases at the −2, 0, and +1 through +4 positions as judged by PSPA analysis (Fig 4A). Furthermore, these mutants inverted their respective preferences for PAKtide and MSTtide.

We focused on the PAK4^M4^ mutant because it appeared to have a more completely reprogrammed substrate specificity in comparison to MST^P4^. PAK4^M4^ still had lower activity than WT PAK4, which would complicate its use in cell-based experiments because loss of function could be attributable to reduced activity rather than altered specificity. We observed that PAK4^M4^ less efficiently underwent autophosphorylation within its activation loop (S7 Fig), a critical step in activating the kinase. Because its activation loop phosphorylation site conforms well to the PAK4 consensus sequence (S7A Fig), it is likely that reduced autophosphorylation is a consequence of the reprogrammed specificity of PAK4^M4^. Incorporation of a phosphomimetic mutation at the activating site (S474E) better normalized the activity of PAK4^M4^ relative to PAK4^S474E^ (to 63%; S7C Fig). Because they had similar activity on their respective favored substrates, we proceeded to compare their ability to function in PAK- and MST-dependent processes.

**Fig 6:**
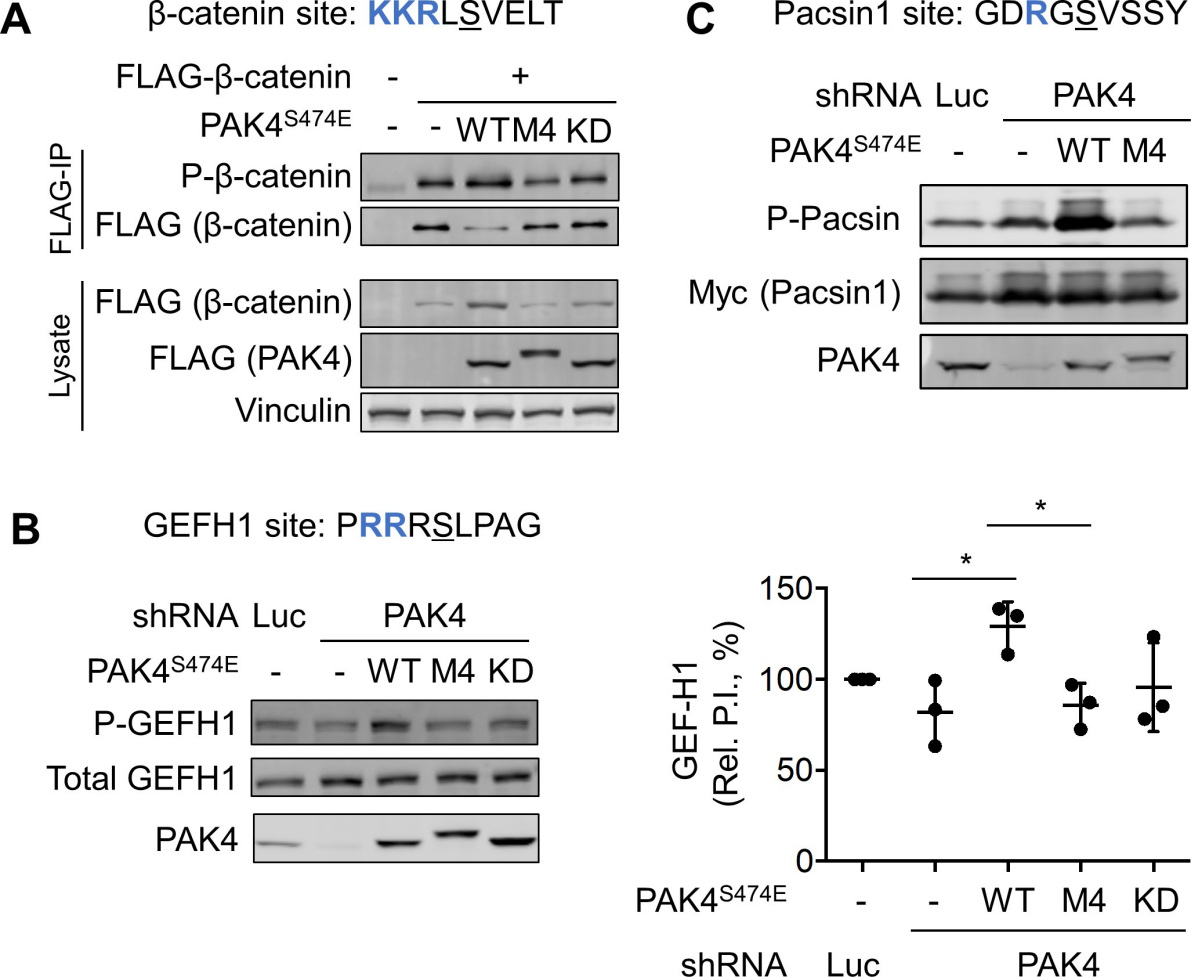
Catalytic site interactions are required for PAK4 to target protein substrates in cells. (A) HEK293A cells were co-transfected with plasmids expressing FLAG-epitope–tagged β-catenin and PAK4^S474E^ with the additional mutations as indicated. Following FLAG immunoprecipitation from cell lysates, equal amounts of purified β-catenin were subjected to immunoblotting to detect phosphorylation at Ser675. (B) Panc1 cells expressing a doxycycline-inducible shRNA directed to PAK4 or a nontargeting control shRNA were transfected with plasmids expressing the indicated forms of PAK4^S474E^. Phosphorylation of endogenous GEF-H1 at Ser886 was assessed by immunoblotting with a phosphospecific antibody. The phosphorylation index (ratio of phospho-GEF-H1 to total GEF-H1) was quantified and normalized to the empty vector control (*n* = 3, error bars indicate SD). Empty vector, PAK4^S474E^, and PAK4^M4/S474E^ signals were compared with unpaired *t* tests (*p < 0.05). Numerical data are provided in S2 Data. (C) The Panc1 cell lines used in (B) were co-transfected with plasmids expressing Myc-epitope–tagged Pacsin1 and the indicated PAK4^S474E^ mutants, and Pacsin1 phosphorylation at Ser346 was analyzed by immunoblotting. GEF-H1, Rho guanine nucleotide exchange factor H1; HEK, human embryonic kidney; IP, immunoprecipitation; KD, kinase-inactive mutant; Pacsin1, Protein kinase C and casein kinase substrate in neurons protein 1; PAK, p21-activated kinase; P.I., phosphorylation index; P-, phospho-; shRNA, short hairpin RNA; WT, wild type.

We initially examined whether PAK4^M4^ was capable of phosphorylating a set of established PAK4 substrates in cultured cells, including β-catenin, Rho guanine nucleotide exchange factor H1 (GEF-H1), and the Fer/Cip4-Bin-Amphiphysin-Rvs (F-BAR) protein Protein kinase C and casein kinase substrate in neurons protein 1 (Pacsin1). We found that coexpression in human embryonic kidney (HEK)293A cells with PAK4^S474E^, but not other forms of PAK4, caused β-catenin to accumulate to higher levels than when expressed alone (Fig 6A), possibly because of its stabilization by phosphorylation at Ser675. To determine relative levels of β-catenin Ser675 phosphorylation in cells expressing various forms of PAK4, we isolated the protein from cell lysates and analyzed equal quantities by immunoblotting. In keeping with our in vitro kinase assays, we found that PAK4^S474E^ robustly induced β-catenin phosphorylation at Ser675 in cells (Fig 6A). By contrast, we observed no increase in phosphorylation upon coexpression with PAK4^M4/S474E^ or with kinase-inactive mutant (KD) PAK4 (PAK4^D440N/S474E^). To examine substrate phosphorylation in an endogenous setting, we performed experiments in Panc1 pancreatic cancer cells that express PAK4 to high levels [52]. Silencing PAK4 expression in Panc1 cells caused only a slight reduction of endogenous GEF-H1 phosphorylation, presumably due to compensation from other kinases (Fig 6B). Importantly, re-expression of PAK4^S474E^, but not PAK4^M4/S474E^ or PAK4^KD^, led to elevated GEF-H1 phosphorylation. Because Panc1 cells do not detectably express Pacsin1, we examined phosphorylation of ectopically expressed protein in this system. As with other substrates, PAK4^S474E^ alone robustly enhanced phosphorylation of Pacsin1 (Fig 6C). Given the high levels of substrate phosphorylation induced by PAK4^S474E^, the slightly lower intrinsic kinase activity of PAK4^M4/S474E^ (S7C Fig) is unlikely to underlie its complete inability to phosphorylate substrates in cells. Indeed, PAK4^M4/S474E^ failed to induce Pacsin1 phosphorylation even when expressed to higher levels than PAK4^S474E^ (S8 Fig). Collectively, these results suggest that catalytic site specificity is essential for phosphorylation of at least some authentic PAK4 protein substrates.

**Fig 7:**
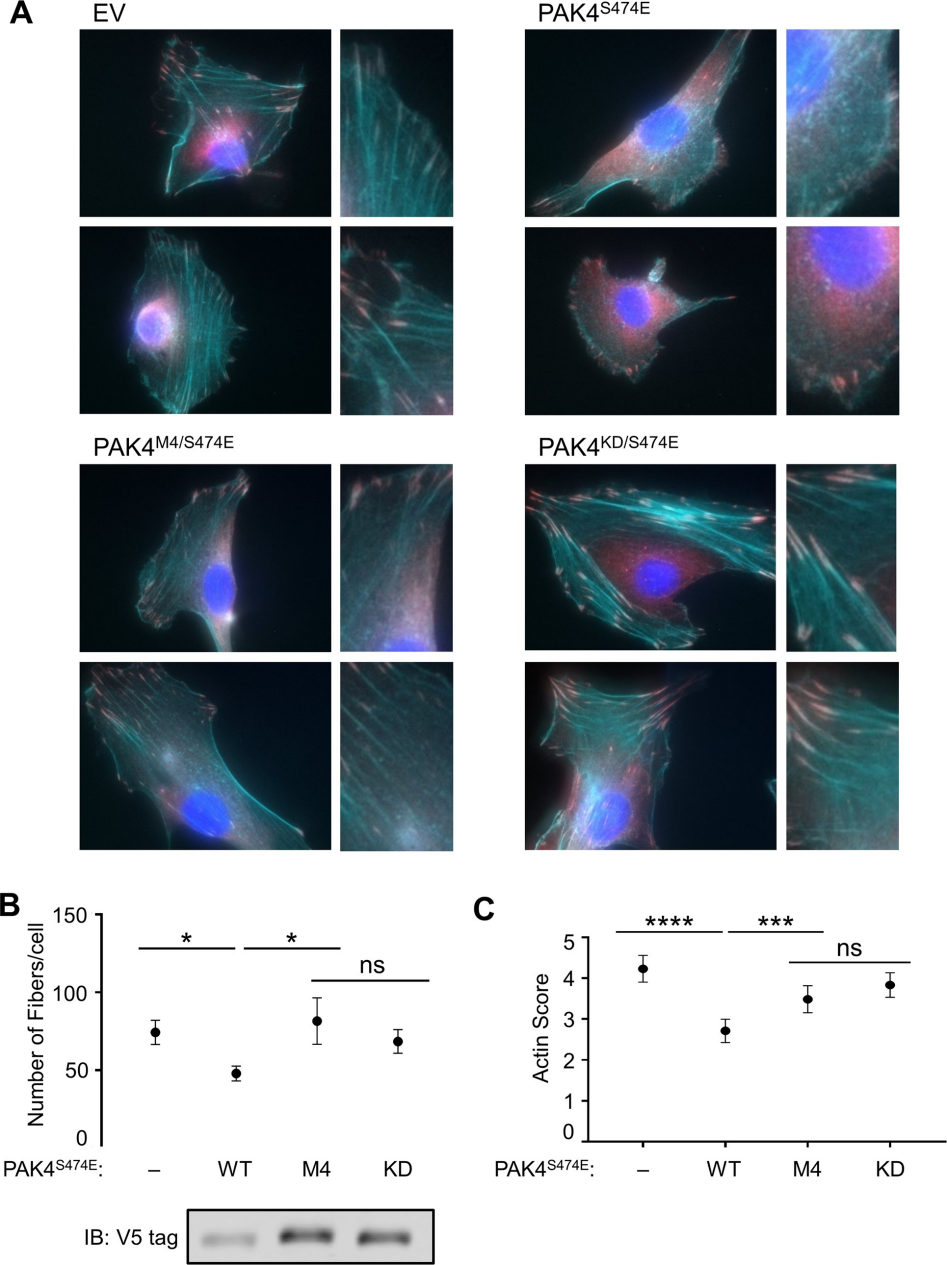
Phosphorylation site specificity is required for actin disassembly by PAK4. (A) NIH-3T3 cells stably expressing the indicated PAK4 mutants were imaged following staining with phalloidin (cyan), α-vinculin (magenta), and DAPI (blue). (B) The number of actin fibers per cell were identified using SFEX software [54] to analyze blinded images. More than 30 cell images were analyzed for each condition. (C) Actin disassembly was scored manually on a 7-point scale in a blinded manner, with higher values indicating more actin fiber disassembly. At least 150 cells across three separate experiments were analyzed per condition. The scores of all four conditions were tested for differences using an ordinary, one-way ANOVA, which was significant (*F* = 16.99, *p* < 0.0001), followed by Fisher’s least significant difference post hoc test (***p < 0.001; ****p < 0.0001; ns, not significantly different at *p* = 0.05). Error bars indicate 95% CI. Cell lysates were immunoblotted to determine expression levels of V5-tagged proteins. Numerical data for (B) and (C) are provided in S2 Data. DAPI, 4′,6-diamidino-2-phenylindole; EV, empty vector; IB, immunoblot; KD, kinase-inactive mutant; PAK, p21-activated kinase; SFEX, stress fiber extractor; WT, wild type.

We next examined the contribution of phosphorylation site specificity to PAK4-dependent cytoskeletal remodeling in fibroblasts. Expression of active PAK4 causes disassembly of actin stress fibers in fibroblasts and other cell types, due at least in part to direct phosphorylation and down-regulation of GEF-H1 [22, 53]. In NIH-3T3 cells expressing PAK4^S474E^, there were fewer actin stress fibers relative to either empty vector or KD (PAK4^D440N/S474E^) control (Fig 7). In contrast, PAK4^M4/S474E^ was not significantly different from the KD PAK4 in promoting reorganization of actin fibers. Taken together, these results indicate that PAK4 phosphorylation site specificity is necessary for regulation of the actin cytoskeleton, a key signaling output of the kinase.

### Catalytic site specificity is sufficient for signaling through the Hippo pathway

We next asked whether phosphorylation site specificity could be sufficient for signaling from STE20 kinases. Multiple STE20 family kinases have roles as upstream regulators of the tumor-suppressive Hippo pathway. This pathway integrates signals from cell–cell contact and G-protein–coupled receptors, leading to decreased cell growth and survival [13]. Canonically, MST1 and MST2 phosphorylate and activate the kinases LATS1 and LATS2, which themselves phosphorylate the transcription factors YAP and TAZ. Phosphorylation of YAP and TAZ induces their nuclear exclusion and proteasomal degradation. In at least some contexts, other STE20 kinases can function in place of MST1 and MST2. For example, combined ablation of genes encoding MST1/2 and six related STE20 kinases was required to fully down-regulate Hippo signaling in HEK293A cells [16, 18]. Notably, the LATS hydrophobic motif site conforms closely to the phosphorylation site motif recognized by GCKi–v group kinases. We therefore examined whether reprogramming the phosphorylation site specificity of PAK4 could allow it to phosphorylate LATS and function in the Hippo pathway.

**Fig 8:**
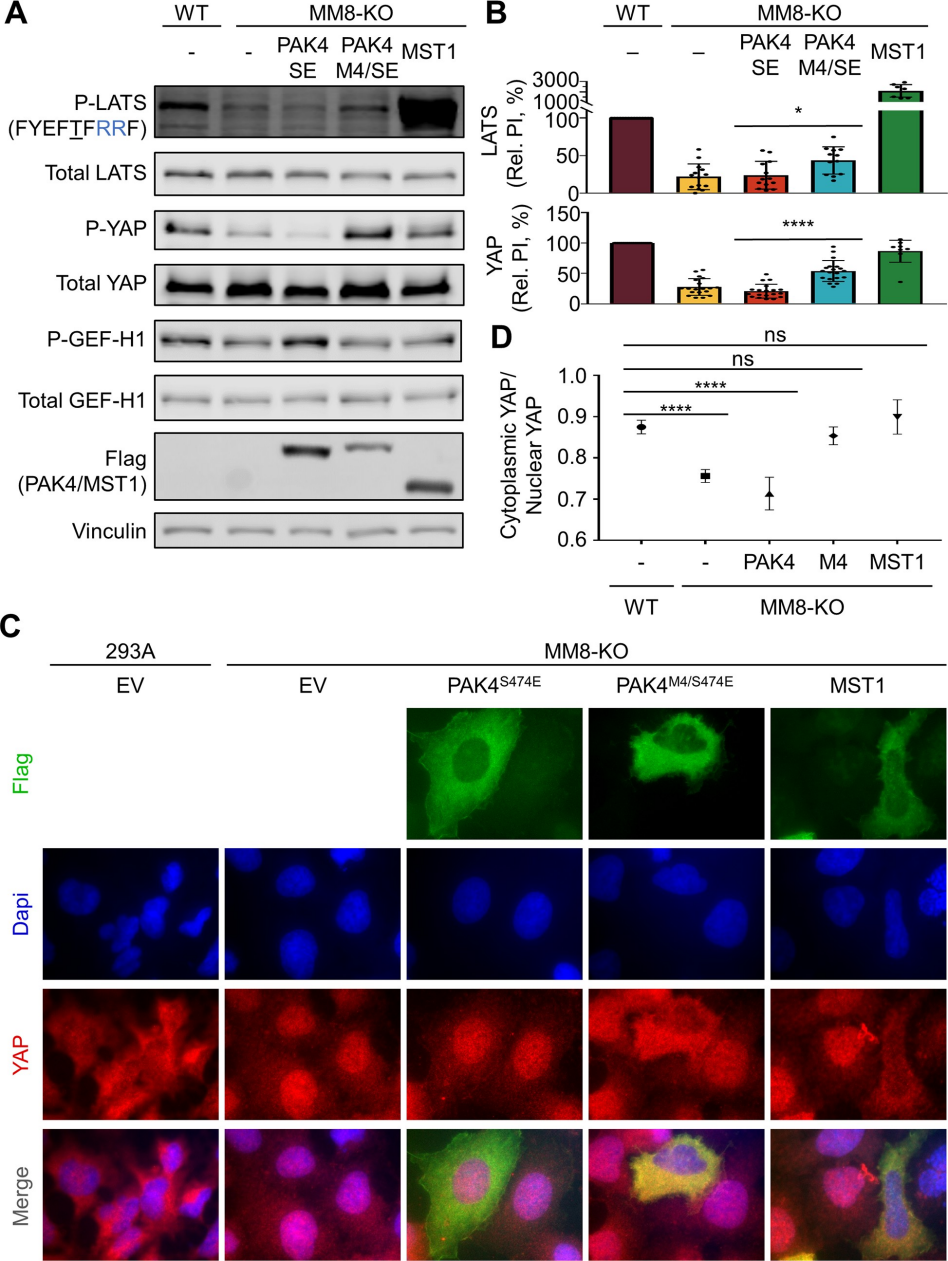
Signaling through the Hippo pathway by re-engineered PAK4. (A) Phosphorylation of LATS and YAP in parental HEK293A (WT) or a derivative lacking eight STE20 family kinases (MM8-KO) were analyzed by immunoblotting following transfection with plasmids expressing the indicated kinases. (B) Quantified Rel. PIs from immunoblots were normalized to the signal from WT cells (*n* ≥ 7, error bars indicate SD). Student *t* tests were used to determine whether the indicated pairs were significantly different from each other (*p < 0.05; ****p < 0.0001). (C) Parental HEK293A or MM-8KO cells transfected with the indicated constructs and serum starved for 60 min were imaged after staining with DAPI (blue) and antibodies to YAP (red) and FLAG epitope (green). (D) Automated scoring (CellProfiler) of YAP nucleocytoplasmic distribution. More than 90 cells were scored for each condition in three replicate experiments. Values were tested for significant differences by an ordinary one-way ANOVA (*F* = 34.06, *p* < 0.0001), followed by Dunnett’s test to compare each condition to the mock transfected parental line (293A) values (****p < 0.0001; ns, not significantly different at *p* = 0.05). Error bars represent 95% CI. Numerical data for (B) and (D) are provided in S2 Data. DAPI, 4′,6-diamidino-2-phenylindole; EV, empty vector; GEF-H1, Rho guanine nucleotide exchange factor H1; HEK, human embryonic kidney; LATS, large tumor suppressor homolog; MST, Mammalian sterile 20 kinase; PAK, p21-activated kinase; P-, phospho-; Rel. PI, relative phosphorylation index; SE, standard error; WT, wild type; YAP, Yes-associated protein.

We confirmed that HEK293A cells lacking these eight kinases (MM-8KO) had substantially reduced Hippo pathway signaling as judged by LATS and YAP phosphorylation, as well as nuclear exclusion of YAP (Fig 8). Expression of PAK4^M4/S474E^ in these cells partially restored LATS phosphorylation, albeit less efficiently than did MST1 (Fig 8A and 8B). As expected, active PAK4^S474E^ mediated GEF-H1, but not LATS, phosphorylation. Though PAK4^M4/S474E^ did not promote complete phosphorylation of LATS, it was sufficient to effect nearly full phosphorylation of a key regulatory site on YAP, suggesting that a low threshold of LATS activity is sufficient for Hippo signaling. A chimeric protein, in which the PAK4^M4/S474E^ catalytic domain replaced that of MST1, was equivalent to PAK4^M4/S474E^ in inducing LATS phosphorylation (S9 Fig), suggesting that other regions of MST1 do not promote the activity of the re-engineered kinase. We also found that MST1 and PAK4^M4/S474E^ promoted cytoplasmic retention of YAP to the same extent, while PAK4^S474E^ was without effect (Fig 8C and 8D). Though there was substantial variability between experiments, we saw no consistent effect of GCKi–v kinase deletion or expression of PAK4 mutants on signaling through other established growth control pathways in these cells (S10 Fig), suggesting that effects on YAP phosphorylation and localization are unlikely to directly involve components of these other pathways. Taken together, these results suggest that the phosphorylation site motif of a STE20 kinase is sufficient for participation in the Hippo signaling cascade.

## Discussion

While mechanisms of substrate targeting have been explored in depth for a number of well-studied kinases, a detailed understanding of phosphorylation site specificity has been lacking for the majority of these enzymes [6, 42]. Here, we have systematically profiled the substrate specificity of STE20 kinases and correlated their sequence preference with specific features within the kinase catalytic domain. For example, we found that mutation of two residues within the KxxN catalytic motif could exchange specificity between PAK4 and MST4 at the −2 position. This observation is consistent with x-ray crystal structures of multiple kinase-peptide complexes, including PKA, in which an Arg residue at the −2 position makes direct contact with an acidic residue within the KxxN motif [55]. Furthermore, mutation of the analogous residue in PKCθ to Ala was reported to reduce selectivity for a −2 Arg residue [45]. Less anticipated was the identification of the β3–αC loop region as a key determinant for selecting basic residues downstream of the phosphorylation site for kinases within and outside of the STE20 family. Previous studies have implicated this region in mediating substrate selectivity. For example, mutating two residues in this loop could exchange the preferences for specific hydrophobic residues at the +2/+3 positions between type I and type II PAKs [35]. In addition, the presence of basic residues in region is at least partly responsible for substrate specificity of casein kinase 2 (CK2) and glycogen synthase kinase-3 (GSK3), which select acidic or phosphorylated amino acids downstream of the phosphorylation site [48, 56]. In addition to being involved in interactions with substrates, the positioning and dynamics of helix αC strongly influences protein kinase catalytic activity [57]. The capacity of this region to act as a general hub for substrate recognition suggests a mechanism by which adopting a more active conformation is dependent on interactions with the bound substrate. Many kinases, including PAKs, have a conserved basic residue at the N-terminus of helix αC that makes direct contact with a phosphorylated Ser or Thr residue in the kinase activation loop to promote kinase activity. By contrast, GCKi–v and PKC isozymes that have a highly acidic β3–αC loop lack this basic residue. The intermolecular interaction between basic residues in the substrate and the acidic β3–αC loop region would appear to be effectively a charge reversal that substitutes for an intramolecular contact. In keeping with this idea, the presence of basic residues C-terminal to the phosphorylation site affected the *k*_*cat*_ rather than *K*_*m*_ value for phosphorylation of a peptide substrate by PKCα, suggesting that engagement of the β3–αC loop by these residues indeed serves to promote catalysis [58].

We note that the residues we chose to exchange between PAK4 and MST4 were identified on the basis proximity to the substrate, as seen in crystal structures of PAK4–peptide complexes. Because crystal structures can only reveal static snapshots of these interactions, we cannot rule out additional contacts that contribute to substrate selectivity. Molecular dynamics simulations, for example, revealed a key specificity-determining residue in nonreceptor tyrosine kinases that was not evident from cocrystal structures with peptides [59]. Furthermore, computational analyses have suggested that residues not directly contacting the substrate can contribute to specificity [60, 61]. Because our mutants often had substantially decreased catalytic activity, these other residues may function to maintain activity in the presence of particular residues in the catalytic cleft.

While the largest distinction we observed in phosphorylation site specificity was between the PAK and GCK subfamilies, we could further categorize the GCK kinases into three groups. The most divergent kinase was TAO2, which we unexpectedly found to select phosphorylated residues at several positions. TAO2 thus appears to recognize substrates that are “primed” by prior phosphorylation at nearby residues, as has been established for kinases such as CK2 and GSK3β. This phenomenon likely explains in part the sequential phosphorylation by TAO2 of two residues within the activation loops of three MAPK kinases (MKK3, 4, and 6). TAO2 initially phosphorylates the more downstream residue, followed by a second residue located four positions upstream [62]. This order of phosphorylation was previously rationalized based on selectivity of TAO2 for an acidic residue at the −5 position, which is mediated by a pair of basic residues located in the kinase αF–αG loop. Our PSPA analysis suggests that this initial phosphorylation event is also guided by a Thr phosphoacceptor, a hydrophobic residue at the +1 position, and an acidic residue at the +2 position. Phosphorylation at this site then primes for phosphorylation at the more upstream site by placing a phosphorylated residue at the +4 position. Such “self-priming” is frequently observed with kinases that prefer phosphorylated amino-acid residues [2] and may generally apply to TAO2 substrates. Notably, a group of direct TAO2 phosphorylation sites identified through chemical genetics [43] all possessed Ser or Thr residues at downstream positions, many of which have been observed to be phosphorylated in phosphoproteomic studies [11].

Comparative analysis of the entire STE20 family allowed us to rationally design mutants that exchange specificity between kinases with divergent phosphorylation site preferences. Previous efforts to re-engineer kinase specificity have typically focused on individual residues that determine specificity at a single position near the phosphorylation site [45, 47, 63, 64]. These studies have provided insight into the structural basis of kinase specificity as well as the impact of kinase mutations occurring during evolution or in human tumors. Here, we have completely re-engineered PAK4 and MST4 to effect more radical changes in specificity. The resulting PAK4 mutant harboring the specificity profile of a GCKi–v kinase was used to investigate the contribution of phosphorylation site specificity to substrate targeting within the STE20 group. Perhaps not surprisingly, this mutant failed to phosphorylate known PAK4 targets and to induce changes to the actin cytoskeleton characteristic of the WT kinase. These results are in keeping with previous observations that kinase mutations causing more subtle perturbations in phosphorylation site specificity can also cause loss of function [46, 47, 65]. PAK4 targets GEF-H1 in part through a noncatalytic domain [53]. Our results suggest that this interaction alone is insufficient to effect GEF-H1 phosphorylation at Ser886, a site that conforms very closely to the PAK4 target motif. Likewise, our kinetic analysis of β-catenin phosphorylation suggests potential docking interactions with PAK4, which could not override the requirement for complementary catalytic site interactions. We note that in many cases, kinase phosphorylation sites match poorly to their target consensus sequences [66, 67]. For example, while some features of the motifs we determined by PSPA are recapitulated in protein substrates (Fig 4B), there are notable exceptions, including a lack of Trp residues at the +1 position for all kinases examined. In these cases, other interactions, either occurring directly to the kinase itself or through scaffold and adaptor proteins, may have a more critical role by effecting substrate recruitment. Alternatively, it may be that selectivity for particular residues is dependent on the surrounding sequence context. Indeed, we found that substitution of the residue at the +1 position in β-catenin with Trp impaired, rather than improved, its phosphorylation rate by PAK4. In many cases it is likely that the phosphorylation site sequence has been selected to be suboptimal. Suboptimal phosphorylation site sequences confer sensitivity to perturbation, which may facilitate selective phosphorylation of more efficient kinase substrates in particular contexts [68–70]. While it is therefore possible that PAK4 substrates exist that are phosphorylated independently of its phosphorylation site motif, the inability of mutant PAK4 to reorganize the actin cytoskeleton suggests that these other substrates are insufficient to mediate at least one major signaling output of this kinase. We also note that in the system we employed, actin stress fiber disassembly required ectopic expression of constitutively active kinase, and we cannot rule out a requirement for catalytic site specificity in other functions dependent on endogenous PAK4.

By contrast to its inability to mediate PAK4 function, we found that the PAK4^M4/S474E^ mutant was able to function in place of GCKs in the Hippo signaling pathway. This observation is consistent with the ability of GCKs from distinct subfamilies to redundantly act in the Hippo pathway despite having different domain architectures and interacting proteins. For example, the canonical Hippo kinases MST1 and MST2 are characterized by a C-terminal coiled-coil region termed the Sav-Rassf-Hpo (SARAH) domain, which associates with Ras-association-domain–containing protein (RASSF) family tumor suppressor proteins and the adaptor protein Salvador (SAV) [14]. By contrast, members of the GCK-I subfamily, which includes multiple additional LATS kinases, have distinct interaction partners including SH3-domain–containing adaptor proteins and bind to some substrates and regulators through C-terminal citron homology domains of unknown structure. The only similarity between the two subfamilies is that they share a kinase domain having identical specificity, suggesting that catalytic site interactions are sufficient to mediate at least some level of signaling through the pathway. We did observe that LATS was phosphorylated weakly by PAK4^M4^ in comparison to authentic Hippo kinases, suggesting that catalytic site interactions alone do not provide maximal activity. We note that PAK4^M4^ had equivalent LATS kinase activity as a chimeric protein in which the MST1 catalytic domain was replaced with that of PAK4^M4^. This result argues against the involvement of noncatalytic domains and interaction partners such as SAV in promoting LATS phosphorylation. The low level of LATS phosphorylation by PAK4^M4^ could reflect the intrinsically low catalytic activity of PAK4 in comparison with GCKi–v kinases [46]. Another potential contributing factor is that MST kinases are reported to autophosphorylate at sites outside the catalytic domain to induce interaction with the LATS adaptor protein Mps1 binder kinase activator-like 1 (MOB1) [71, 72]. In this case, inefficient autophosphorylation by PAK4^M4^ or the absence of the critical MOB1 binding sites could attenuate its ability to phosphorylate LATS. We cannot exclude a role for protein localization in promoting LATS phosphorylation, and we note that both PAK and MST kinases are found at least in part at the plasma membrane, which may be important for signaling through the pathway.

In summary, we have shown that catalytic site is at least to some extent both necessary and sufficient for the signaling output of some kinases in the STE20 family. These observations may seem at odds with the wealth of prior data suggesting important roles for docking and adaptor protein interactions. However, in other systems, specific elements of kinase–substrate interactions appear to confer robustness to perturbation rather than serve as a binary switch for substrate selection [68, 69, 73]. Furthermore, it is likely that an important role for noncatalytic site interactions is to restrict the specificity of kinases, preventing potentially deleterious phosphorylation of irrelevant proteins. We suggest that these principles of kinase–substrate recognition are thus likely to have more general relevance to other eukaryotic kinase groups outside of the STE20 family.

## Materials and methods

### Plasmids

Primers for cloning and mutagenesis are listed in S1 Table. Gateway donor vectors for PAK4 (Uniprot O96013-1) and TAO2 catalytic domain (Uniprot Q9UL54-2, residues 1–320) were made by PCR amplification of their respective cDNAs and BP recombination into pDONR221. Donor vectors for the following kinases were obtained from the human ORFeome collection [74]: GCK (Uniprot Q12851-2), HGK (Uniprot O95819-5), HPK1 (Uniprot Q92918-2), KHS2 (Uniprot Q8IVH8-3), MINK (Uniprot Q8N4C8-1), MST2 (Uniprot Q13188-1), MST3 (Uniprot Q9Y6E0-2), LOK (Uniprot O94804), and PKCβ (Uniprot P05771-2). Mammalian transient expression constructs for WT and mutant kinases were made by LR recombination into the Gateway destination vector (pV1900) derived from pCMV-Sp6 and encoding a C-terminal 3× FLAG epitope tag, with the exception of PAK4, which was made by Gibson assembly into pcDNA3-FLAG, and MST1 (pcDNA3-Flag-mMST1, Addgene #1965; Watertown, MA, USA), which was generated by the laboratory of Joseph Avruch. The mammalian expression vector pcDNA3-FLAG-β-catenin was generated by the laboratory of Eric Fearon and obtained from Addgene (#16828), and the expression vector for Myc-tagged Pacsin1 was from the laboratory of Jeffrey Peterson. PAK4 lentiviral expression constructs were made by Gateway recombination from pDONR221 into pLX304 (Addgene #25890, laboratory of David Root). Bacterial expression constructs expressing 6× His-tagged PAK4 and MST4 catalytic domains were previously described [22, 46]. WNK1 (1–661), OSR1, and MO25α in the pGEX-6P-1 backbone were from the Division of Signal Transduction Therapy, University of Dundee (Dundee, Scotland). For bacterial coexpression, the Ser382 codon of WNK1 was mutated to TAG (WNK1-S^P^382), and the ampicillin resistance marker of the OSR1 was replaced with a zeocin resistance marker cassette. The bacterial expression construct for Yeast Sps1/Ste20-related Kinase 1 (YSK1) catalytic domain (residues 2–293) was generated by PCR-based subcloning of the full ORF into pCDF, followed by introduction of an appropriately placed stop codon. The bacterial expression vector producing N-terminally hexahistidine-tagged mouse β-catenin was generated by subcloning the full-length ORF into a modified pET32 plasmid.

Point mutations were introduced using the QuikChange protocol (Stratagene, San Diego, CA, USA). All kinase-inactive mutant controls substituted the catalytic Asp in the HRD×KxxN motif with Asn. PAK4 specificity altering mutations were combinations of the following: β3–αC loop region, R355–L362→EEAEDEIED; KxxN, S443A/D444A; DFG+1, F461V; activating mutation, S445N. MST4 mutants were combinations of: β3–αC loop region, E58–D66→RKQQRREL; KxxN, A147S/A148D; DFG+1, V165F. The PAK4–MST1 chimera constructs comprise residues 109–426 of PAK4 (Uniprot O96013-2) followed by 322–487 of MST1 (Uniprot Q9JI11-1) and were constructed by overlap extension PCR, followed by Gateway recombination into pV1900. PAK4 constructs for expression in shPAK4-expressing cells were rendered shRNA resistant by incorporating three silent point mutations at the target site. β-Catenin plasmids for bacterial expression included two point mutations to remove minor PAK4 phosphorylation sites (S552A/T556A) and combinations of the following: +1W (V676W), MST motif (K672–S680→GYNTVRRKK), and phosphorylation resistant controls (S675A or T675A).

### Protein production and purification

The catalytic domains of PAK4 [46], MST4 [46], SLK [36], TNIK [42], YSK1, PAK2 [35], PAK6 [40], and CDC42 [22] were expressed in bacteria as previously reported, and the YSK1 catalytic domain was purified as described for MST4. MYO3A and MYO3B, containing the kinase motor and two calmodulin binding sites as well as PAK1, were expressed in Sf9 insect cells as previously reported [35, 75].

Active preparations of OSR1 were prepared by coexpressing WNK1-S^P^382 with WT OSR1 in EcAR7 [76] cells containing SepOTS [77], following general procedures as described previously [78]. For purification of OSR1 and MO25α expressed as GST fusion proteins, induced bacterial cell pellets from 100 mL cultures were resuspended in 5 mL of bacterial lysis buffer (50 mM Tris-HCl [pH 7.4], 500 mM NaCl, 0.5 mM EDTA, 0.5 mM EGTA, 5 mM DTT, 1 mg/mL lysozyme, 50 mM NaF, 1 mM NaVO_4_, 10% glycerol, Roche protease inhibitor tablet), incubated on ice for 30 min, and sonicated. Lysates were clarified by two rounds of centrifugation at 22,000 × *g* for 15 min at 4°C. The clarified lysate was transferred to 200 μL bed volume of Glutathione Hi-Cap Matrix (Qiagen, Valencia, CA, USA) pre-equilibrated in lysis buffer and rotated for 1 hour at 4°C. The slurry was centrifuged at 500 × *g*, 5 min 4°C, and the pellet was resuspended and transferred to a column and washed with 6 mL of lysis buffer without lysozyme or protease inhibitors. Proteins were eluted by rotation with 200 μL of the same buffer containing 20 U PreScission protease (GE Healthcare, Pittsburgh, PA, USA) at 4°C overnight with agitation, followed by washing with an additional 400 μL of lysis buffer. Eluted fractions were pooled, concentrated, and buffer exchanged into a storage buffer (50 mM Tris/HCl [pH 7.4], 150 mM NaCl, 1 mM DTT, 20% glycerol) using a 0.5 mL Amicon ultra centrifugal filter (Millipore, Billerica, MA, USA), and the protein was stored at −20°C. Protein concentrations of OSR1, MO25α, PAK4, and MST4 were determined using BSA standards by SDS-PAGE and Coomassie staining.

FLAG-epitope–tagged kinases were produced by polyethyleneimine (PEI) transfection of HEK293T cells and purified through batch FLAG affinity chromatography. Low-passage cells were seeded into 2 × 10 cm plates (9 × 10^5^ cells/plate) and incubated overnight. Each plate was transfected with 15 μg plasmid DNA and 45 μL of 1 mg/mL PEI (PEI) as previously described [79]. After incubating for 40 hours, cells were washed twice with ice-cold PBS and 1 mL mammalian cell lysis buffer (150 mM NaCl, 20 mM Tris [pH 7.5], 1 mM EGTA, 1 mM EDTA, 1% Triton X100, 2.5 mM sodium pyrophosphate, 1 mM Na_3_VO_4_, 1 mM DTT, 1 mM PMSF, 1 mM β-glycerophosphate, 10 μg/mL leupeptin, 2 μg/mL pepstatin, 10 μg/mL aprotinin) was added to each plate. Lysates were scraped into 1.5-mL tubes and incubated on ice for 10 min. After clarification in a 4°C microfuge, the supernatant was mixed with 75-μL anti-FLAG M2 beads (Sigma Aldrich, St. Louis, MO, USA) and rotated for 2 hours at 4°C. Beads were centrifuged and washed twice with cell lysis buffer, twice with wash buffer (50 mM HEPES [pH 7.4], 100 mM NaCl, 1 mM DTT, 5 mM β-glycerophosphate, 0.1 mM Na_3_VO_4_, 0.01% Igepal CA630, 10% glycerol). Protein was eluted into 250 μL wash buffer containing 0.5 mg/mL 3× FLAG Peptide (APExBIO, A6001; Houston, TX, USA) at 4°C for 1 hour. The supernatant was filtered to remove beads, and kinase concentration was determined by SDS-PAGE and Coomassie staining using a BSA standard curve.

For production of full-length β-catenin, 200 mL bacterial cultures were induced and grown overnight at 16°C. Pellets were resuspended in 5 mL β-catenin lysis buffer (20 mM Tris [pH 8.8], 140 mM NaCl, 10 mM imidazole [pH 7.4], 10 μg/mL pepstatin A, 10 μg/mL leupeptin, 3 mM β-mercaptoethanol, 0.4% Igepal CA630, 13 mM MgCl_2_, 1 mM PMSF, 200 μg/mL lysozyme). Cell suspensions were sonicated, DNAse I was added to 0.03 U/μL, and lysates were rotated at 4°C for 30 min. After clarification, the lysates were rotated with immobilized metal affinity resin (Talon, Takara, Kusatsu, Japan), at 4°C for 30 min. Beads were transferred to a column and washed twice with 5 mL PBS/0.5% Igepal CA630, once with 4 mL Talon wash buffer (20 mM Tris, 500 mM NaCl, 10 mM imidazole [pH 7.4]), and eluted in 3 mL elution buffer (20 mM Tris, 140 mM NaCl, 250 mM imidazole [pH 7.4]) with gravity flow. Concentrated fractions were pooled and dialyzed into dialysis buffer (10 mM HEPES [pH 7.4], 100 mM NaCl, 10% glycerol, 1 mM DTT) overnight at 4°C. Protein concentration and purity were determined by staining of an SDS-PAGE gel with Coomassie brilliant blue and comparison to BSA standards.

### PSPA assay

The PSPA (Kinase Substrates Library, Groups I and II, Anaspec, Fremont, CA, USA) consists of 198 peptide mixtures having the general sequence Y-A-X-X-X-X-X-S/T-X-X-X-X-G-A-K-K(biotin), in which S/T is an equimolar mixture of Ser and Thr and eight of the nine X positions are a degenerate mixture of 17 residues (all but S, T, and C). In each peptide mixture, one X position is fixed as one of the standard 20 amino acids, pThr, or pTyr. The library also contains three peptide mixtures that are degenerate at all X positions but have either Ser, Thr, or Tyr fixed at the central position to determine phosphoacceptor preferences.

The reported PSPA assay [38, 39] was modified as follows. Buffer (recipes below) was added to 1,536 well reaction plates (2 μL per well) using a Mantis nanodispenser (Formulatrix, Bedford, MA, USA). Aqueous peptides (250 nL) were transferred from 384-well stock plates to the reaction plates using a Mosquito liquid handler (TTP Labtech, Melbourn, UK) to a final concentration of 51 μM. Kinase and ATP in reaction buffer (200 nL per well) were added by Mantis to a final ATP concentration of 45 μM with 0.027 μCi/μL [γ-^33^P] ATP. The reaction plate was sealed, centrifuged briefly, and incubated at 30°C for 2 h. Reactions were spotted onto a streptavidin membrane (Promega, Madison, WI, USA), which was washed, dried, and imaged as described previously [38, 39]. Spot intensities were quantified using Quantity One software (Bio-Rad, Hercules, CA, USA), and normalized by dividing by the average intensity of all spots in the same sequence position. Normalized values of replicates were averaged, converted to a log_2_ scale, and presented as heat maps (created in Excel) or enoLOGOS [44].

PSPA reactions for most kinases were run in universal kinase buffer (50 mM Tris [pH 7.5], 10 mM MgCl_2_, 2 mM MnCl_2_, 1 mM DTT, 0.1 mM EGTA, in 0.1% Tween 20) including 820 nM PKI (Sigma Aldrich). The following buffers including 0.1% Tween 20 were used for the indicated kinases: HGK and OSR1 (50 mM Tris [pH 7.5], 10 mM MgCl_2_, 1 mM DTT, 0.1 mM EGTA), MINK (50 mM HEPES [pH 7.4], 10 mM MgCl_2_, 1 mM DTT, 0.1 mM EGTA), MST4 (50 mM Tris [pH 7.5], 10 mM MgCl_2_, 1 mM DTT), PAK4 (50 mM HEPES [pH 7.4], 10 mM MgCl_2_, 12.5 mM NaCl, 1 mM MnCl_2_, 1 mM DTT, 0.1 mM EGTA), PKCβ (50 mM Tris [pH 7.4], 10 mM MgCl_2_, 1 mM DTT with 20% v/v PKC lipid activator). Kinase concentrations ranged from 10–37 nM with the exception of MST4 (320–660 nM), PAK4 (190 nM–2.1 μM), WT PKCβ (6 nM), and PKCβ^3A^ (60 nM). OSR1 reactions included 82 nM GST-MO25. Each kinase was assayed twice, and correlation constants (R^2^) for position-normalized data between replicate runs were >0.6 for all but MST3, which was run two additional times.

### Peptide kinase assays

Peptide substrates were synthesized at the Tufts University Core Facility and purified by HPLC. Kinase, reaction buffer, and peptide (15 μM for PAK4 and 20 μM for all other kinases) were mixed and reactions were started by addition of ATP to a final concentration of 10 μM with 0.05 μCi/μL [γ-^33^P] ATP at a final volume of 20 μL. Reactions were incubated at 30°C, and 5 μL aliquots were taken at 5–7 min intervals, spotted onto P81 phosphocellulose paper filters, and quenched in 75 mM phosphoric acid. Filters were washed 3 × 5 min with 75 mM phosphoric acid, rinsed briefly with acetone, dried, and quantified by scintillation counting. Reaction buffers were the same as used for peptide library assays except that Tween 20 was excluded. PAK1 and PAK2 were assayed in MINK buffer containing 100 nM GTP-γ-S–loaded CDC42. MYO3A and MYO3B were assayed in (10 mM imidazole [pH 7.4], 50 mM KCl, 1 mM EGTA, 1 mM MgCl_2_, 1 mM DTT). Kinase reactions were performed at least three times.

### β-Catenin kinase assays

Full-length PAK4 and full-length β-catenin were assayed in PAK4 buffer and initiated by addition of [γ-^33^P] ATP (10 μM, 0.05 μCi/μL). For steady-state reactions, the PAK4 concentration was 10 nM, and β-catenin concentration was 5 μM. Samples were taken at 5, 10, and 15 min. For single turnover reactions, PAK4 concentration was 500 nM, β-catenin substrates were 1.5 μM, and samples were taken at 5, 10, 15, 20, 30, 60, 90, 120, 150, 240, and 300 seconds. For both steady-state and pre-steady–state experiments, aliquots taken at each time point were quenched with SDS-PAGE loading buffer, boiled, and fractionated by SDS-PAGE. Gels were stained with Coomassie brilliant blue, dried, and exposed overnight to a phosphor storage screen (Kodak, Rochester, NY, USA). Screens were scanned using a phosphorimager (Bio-Rad), and band intensities were quantified using Quantity One software (Bio-Rad).

### Analysis of PAK4 substrate phosphorylation in cultured cells

Panc1 pancreatic cancer cells harboring inducible shRNAs were generated by lentiviral transduction as follows. Lentiviral particles containing inducible shRNA were produced in low-passage HEK293T cells by PEI co-transfection of dR8.91, VsV-G, and PAK4-targeting pTRIPZ #395103 (mature antisense: TGAAGAGCAGCTCGCGCCT; Dharmacon, Lafayette, CO, USA) or luciferase-targeting control vector in a 10:1:10 ratio. Supernatants were collected at 24 and 36 hours post-transfection. Panc1 cells were infected with lentivirus at an MOI of approximately 1 in the presence of 8 μg/mL polybrene. After 24 hours, infected cells were selected with 2 μg/mL puromycin. After 64 hours selection, cells were maintained in 1 μg/mL puromycin, and knockdown was induced with 500 ng/mL doxycycline at least 7 days prior to the experiments below.

To assess phosphorylation of PAK4 substrates, Panc1 cells expressing either shPAK4 or shLuc or HEK293A cells were transfected with the indicated PAK4 expression plasmids either alone or in combination with a 3-fold excess of the indicated substrate expression vector (unless otherwise indicated) using Lipofectamine 2000 as recommended by the manufacturer. Media were exchanged 6 hours later, and cells were incubated for an additional 20 hours (HEK293A cells) or 40 hours (Panc1 cells). For Panc1 cells, media was then aspirated, and cells were washed once with serum-free media and incubated in media containing 0.1% FBS for 2 hours prior to lysis. Cells were transferred to ice, washed once with cold PBS, and extracted into mammalian cell lysis buffer (defined above) lacking DTT. Following incubation for 10 min on ice, lysate was cleared by microcentrifugation (10 min, 4 ºC). Cleared lysates were analyzed by BCA protein assay (Thermo Fisher Scientific, Waltham, MA, USA), and equal amounts of protein were analyzed by SDS-PAGE followed by immunoblotting. FLAG-β-catenin was isolated from HEK293A cell lysates as described above for kinase purification, and relative quantities of recovered protein were determined by immunoblotting samples with FLAG antibody (Sigma M2, #F3165; Sigma Aldrich). Samples containing equal amounts were reanalyzed by immunoblotting with the indicated antibodies. Antibodies used were raised against the following: GEF-H1 pSer886 (Cell Signaling Technology #14145; Danvers, MA, USA), GEF-H1 (Cell Signaling Technology #4076), Pacsin1 pSer346 (Millipore #ABS39), Myc epitope (Cell Signaling Technology 9B11, #2276), β-catenin pSer675 (Cell Signaling Technology #9567), vinculin (Sigma #V9131), and PAK4 (BD Pharmingen, E40-883; Franklin Lakes, NJ, USA). Membranes were imaged using fluorophore-conjugated secondary antibodies (Alexa Fluor 680 goat anti-rabbit IgG and IRDye 800CW donkey anti-rabbit IgG, both at 1:10,000 dilution) on an Odyssey CLx imager (LI-COR, Lincoln, NE, USA).

### Analysis of actin cytoskeleton

Lentivirus expressing PAK4 variants was produced by co-transfecting HEK293T cells with the corresponding pLX304 vector, as well as psPAX2 and VsV-G packaging and envelope plasmids. NIH-3T3 cells were infected by treatment with lentivirus with 8 μg/mL polybrene for 24 h. Cells were then exchanged into fresh media and selected with blasticidin (5 μg/mL for 8 days and in 2.5 μg/mL thereafter). To visualize actin fibers, cells were blinded and sparsely plated onto coverslips in 24-well plates (5,000 cells/well). After 24 hours, cells were washed twice with PBS and fixed in 4% paraformaldehyde for 15 min at room temperature (RT). Cells were washed three times with PBS and permeabilized with 0.1% Triton X100 for 30 minutes at RT. Coverslips were blocked in blocking buffer (1% BSA, 50 mM NH_4_Cl, 0.1% Triton X100 in PBS) for 1 hour and then incubated with an anti-vinculin antibody (Sigma #V9131; Sigma Aldrich) diluted 1:5,000 in PMZ buffer (0.2% BSA, 50 mM NH_4_Cl, 0.1% Triton X100 in PBS) for 1 h. After three PBS washes, coverslips were incubated with Alexa Fluor 568 goat anti-mouse antibody (1:500, Invitrogen A11004; Carlsbad, CA, USA) and Alexa Fluor 647 phalloidin (1:500, Invitrogen A22287) in PMZ buffer for 30 min in a dark, humidified chamber. Coverslips were washed three times with PBS and mounted onto slides using ProLong Diamond mounting media containing 5 μg/mL 4′,6-diamidino-2-phenylindole (DAPI, Invitrogen) and dried overnight in the dark. Cells were imaged at 100× and 40× magnification using a Nikon Eclipse Ti-S microscope (Nikon, Tokyo, Japan), and at least 80 fields with individual cells were captured for each cell line. The entire procedure was performed a total of three times with two different infections. Images were randomly selected from each condition and image set, and blindly scored for actin disassembly using a 7-point scale, with lower values indicating a less extensive stress fiber network. The number of fibers per cell was also quantified in blinded images using SFEX for automated fiber identification [54].

### Hippo pathway activation assays

Parental HEK293A and MM-8KO cells lacking MST1, MST2, KHS2, GCK, HPK1, HGK, TNIK, and MINK [16] were obtained from the laboratory of Kun-Liang Guan. Cells were transfected with vectors expressing the indicated kinases using Lipofectamine 2000 as recommended by the manufacturer. After 16 hours, cells were chilled on ice, washed once with ice-cold PBS, and then mechanically released from the plate into PBS. Cells were gently pelleted at 4°C and suspended in 100 μL of cell lysis buffer (see above). Lysates were clarified by centrifugation, and 4× SDS-PAGE loading buffer was added to the supernatant. Samples were boiled, fractionated by SDS-PAGE, and transferred to PVDF membranes. Membranes were probed with primary antibodies listed above and others from Cell Signaling Technology (LATS1 pThr1079 #8654, YAP pSer127 #4911, YAP #4912, Akt pSer473 D9E #4060, AKT #9272, phospho-ERK #4370, ERK #9102, S6K1 pThr389 #9205, S6K1 #9202), followed by the appropriate secondary antibodies, and imaged on the Odyssey CLx (LI-COR). The phosphorylation index was determined by dividing the phospho-signal to total signal, and samples were normalized to the signals from HEK293A parental cells transfected with empty vector. Experiments were performed at least three times.

For YAP localization experiments, 2.5 × 10^5^ cells were plated in each well of a 12-well plate. After 24 hours, cells were transfected in duplicate with the indicated vectors in a blinded manner using Lipofectamine 2000. After 3–4 hours, cells were trypsinized, and 1.0 × 10^5^ cells were plated onto fibronectin-coated coverslips and incubated overnight. Cells were serum starved for 1 hour before fixation with 4% paraformaldehyde in PBS for 15 min at RT. Immunofluorescence labeling was performed as for actin stress fiber visualization using anti-YAP (Santa Cruz Biotechnology H-125, sc-15407; Dallas, TX, USA) and anti-FLAG (Sigma, M2; Sigma Aldrich) antibodies. Coverslips were imaged at 40× and 100× using a Nikon Eclipse Ti-S microscope (Nikon), with at least 20 FLAG-positive fields captured for each transfection. Images were also analyzed for YAP localization using CellProfiler software [80], using DAPI and FLAG staining to define the nucleus and whole cell, respectively. Unblinding of samples occurred subsequent to automated analysis. This experiment was performed five times.

## Supporting information

S1 Fig Dendrogram of the human STE20 kinase family. Alternative names and subfamily notation as originally proposed by Dan and colleagues [8] are shown. GCK, germinal center kinase; HGK, HPK/GCK-like kinase; HPK1, Hematopoietic progenitor kinase 1; KHS, Kinase homologous to SPS1/STE20; LOK, Lymphocyte-oriented kinase; MINK1, Misshapen-like kinase 1; MST, Mammalian sterile 20 kinase; MYO3, myosin-III; NRK, NIK-related protein kinase; OSR1, Oxidative stress-responsive 1; PAK, p21-activated kinase; SLK, STE20-like kinase; SPAK, STE20/SPS1-related proline-alanine-rich protein kinase; STRAD, STE20-related kinase adapter protein; TAO, thousand and one amino acid kinase; TNIK, Traf2 and NCK-interacting protein kinase; YSK1, Yeast Sps1/Ste20-related Kinase 1.
(TIF)

S2 Fig Reproducibility of semiautomated PSPA assay format. (A) Comparison of the Log_2_ transformed, normalized values for MST4 analyzed using the fully manual PSPA method [81] and the semiautomated method. (B) Comparison of the Log_2_ transformed values from two separate analyses of cyclic PKA using the semiautomated PSPA method. (C) Correlation between normalized values in two replicate PSPA assays for each STE20 kinase analyzed using the semiautomated method. Average R^2^ values for all replicates were 0.75 ± 0.14, with most showing R^2^ > 0.6. For the outlier kinase with the lowest correlation (MST3), two additional PSPA assays were performed. Error bars indicate 95% CIs. Numerical values used to generate all graphs are provided in S3 Data. MST, Mammalian sterile 20 kinase; PKA, cAMP-dependent protein kinase; PSPA, positional scanning peptide array.
(TIF)

S3 Fig PSPA data for additional STE kinases. Heat maps and sequence logos were generated from PSPA data averaged from at least two experiments as in Fig 1. PSPA, positional scanning peptide array.
(TIF)

S4 Fig Motif clustering analysis and PSPA data for both previously and newly analyzed STE20 kinases. Single linkage hierarchical clustering of PSPA data was performed using Spearman rank correlation (cluster 3.0). Before clustering analysis, PSPA data were Log_2_ transformed (with −3 being the lowest allowed value), and Ser/Thr data were removed from all but the 0 position. Logos were generated from positive PSPA selections using enoLOGOS [44]. PSPA, positional scanning peptide array.
(TIF)

S5 Fig Evolutionary conservation of PAK4 and MST4 specificity-determining residues. MST, Mammalian sterile 20 kinase; PAK, p21-activated kinase.
(TIF)

S6 Fig Effect of +1 V to W substitution on β-catenin phosphorylation by PAK4. Kinase assay with full-length WT and +1W (V676W) β-catenin under (A) Michaelis–Menten and (B) single turnover conditions. Data used to generate graphs are provided in S3 Data. PAK, p21-activated kinase; WT, wild type.
(TIF)

S7 Fig Reduced activity of the PAK4^M4^ mutant is normalized by a phosphomimetic mutation. (A) The PAK4 activation loop phosphorylation site sequence conforms to the WT PAK4 recognition motif but is likely to be disfavored by PAK4^M4^. (B) Bacterially expressed PAK4^M4^ catalytic domain has reduced activation loop phosphorylation relative to its WT counterpart. (C) Peptide kinase assays on respective preferred substrates show that introduction of the activation loop phosphomimetic S474E mutation improves the activity of full-length PAK4^M4^ relative to WT PAK4 (*n* = 3, error bars represent SD). Data used to generate the graphs are provided in S3 Data. Kinases were expressed and purified from HEK293T cells. HEK, human embryonic kidney; PAK, p21-activated kinase; WT, wild type.
(TIF)

S8 Fig Phosphorylation of Pacsin1 upon coexpression with varying levels of PAK4 mutants. Panc1 cells expressing inducible shRNA directed to PAK4 or luciferase were co-transfected in 6-well plates with an expression plasmid for Myc–Pacsin1 and increasing amounts of plasmids expressing the indicated PAK4 mutant. Each well received 3 μg of Pacsin1 plasmid and 1 μg total of empty vector mixed with PAK4 expression vector. Quantities of PAK4 vector ranged from 0.2 μg to 1.0 μg. After incubation for 40 hours and serum starvation, cell lysates were prepared and immunoblotted with the indicated antibodies. Pacsin1, Protein kinase C and casein kinase substrate in neurons protein 1; PAK, p21-activated kinase; shRNA, short hairpin RNA.
(TIF)

S9 Fig Activation of Hippo signaling by PAK4/MST1 chimeric kinases. (A) Schematic representation of chimeric kinases. The MST1 kinase domain was exchanged with either PAK4^S474E^ (Chim) or PAK4^M4/S474E^ (Chim^M4^). (B) As in Fig 7A, the indicated kinases were transiently expressed in either the parental or MM-8KO 293A cells, and lysates were analyzed by immunoblotting. A representative blot from three independent experiments is shown, along with quantification of the Rel. PIs (%) of the LATS and YAP blots. Differences in LATS activation were tested using a paired *t* test (*p < 0.05, ns = not significantly different at *p* = 0.05). Error bars indicate SD. Data used to generate the graphs are provided in S3 Data. LATS, large tumor suppressor homolog; MST, Mammalian sterile 20 kinase; PAK, p21-activated kinase; Rel. PI, relative phosphorylation index; YAP, Yes-associated protein.
(TIF)

S10 Fig Assessment of signaling through growth control pathways in MM-8KO cells. Changes in the phosphorylation states of established growth pathways in HEK293A parental and MM-8KO cells upon transfection with PAK4^SE^ and MST1 constructs. Cells lysates were immunoblotted for total and phospho-AKT (pS473), phospho-S6K (pT389), and phospho-Erk (pT202/pY204). The phosphorylation index (phosphorylation signal/total signal) was normalized to the 293A parental value for each experiment. Error bars represent SD, and each experiment was performed at least four times. Data used to generate graphs are provided in S3 Data. Erk, Extracellular signal-regulated kinase; HEK, human embryonic kidney; MST, Mammalian sterile 20 kinase.
(TIF)

S1 Data Quantified PSPA data for all newly analyzed kinases. PSPA data were quantified, background subtracted, then normalized by position as previously reported [82]. Replicate normalized data were averaged and are presented as position weight matrices. If normalized values were negative after background subtraction, a value of 0 was used for that position. PSPA, positional scanning peptide array.
(XLSX)

S2 Data Data used to generate Fig 2A, Fig 2B, Fig 4A, Fig 6B, Fig 7B, Fig 7C, Fig 8B and Fig 8D. (XLSX)

S3 Data Data used to generate S2A Fig, S2B Fig, S2C Fig, S6A Fig, S6B Fig, S7C Fig, S9B Fig, and S10 Fig. (XLSX)

S1 Table List of primers used in this study. (PDF)

## Abbreviations

CK2: casein kinase 2
DAPI: 4′,6-diamidino-2-phenylindole
F-BAR: Fer/Cip4-Bin-Amphiphysin-Rvs
GCK: germinal center kinase
GEF-H1: Rho guanine nucleotide exchange factor H1
GSK3: glycogen synthase kinase-3
HEK: human embryonic kidney
HGK: HPK/GCK-like kinase
HPK1: Hematopoietic progenitor kinase 1
JNK: c-Jun N-terminal kinase
KD: kinase-inactive mutant
KHS: Kinase homologous to SPS1/STE20
LATS: large tumor suppressor homolog
LOK: Lymphocyte-oriented kinase
MAPK: mitogen–activated protein kinase
INK1: Misshapen-like kinase 1
MKK: MAPK kinase
MOB1: Mps1 binder kinase activator-like 1
MST: Mammalian sterile 20 kinase
MYO3: myosin-III
NRK: NIK-related protein kinase
OSR1: Oxidative stress-responsive 1
Pacsin 1: Protein kinase C and casein kinase substrate in neurons protein 1
PAK: p21-activated kinase
PEI: polyethyleneimine
PKC: protein kinase C
PKA: cAMP-dependent protein kinase
PSPA: positional scanning peptide array
pThr: phosphothreonine
pTyr: phosphotyrosine
RASSF: Ras-association-domain–containing protein
RT: room temperature
SARAH: Sav-Rassf-Hpo
SAV: Salvador
SFEX: stress fiber extractor
shRNA: short hairpin RNA
SH3: Src homology 3
SLK: STE20-like kinase
SPAK: STE20/SPS1-related proline-alanine–rich kinase
STRAD: STE20-related kinase adapter protein
TAO: thousand and one amino acid kinase
TAZ: transcriptional co-activator with PDZ binding motif
TNIK: Traf2 and NCK-interacting protein kinase
WT: wild type
YAP: Yes-associated protein
YSK1: Yeast Sps1/Ste20-related Kinase 1
