# Supplementary figures and images for: Comprehensive profiling of the STE20 kinase family defines features essential for selective substrate targeting and signaling output
Source: PLoS Biol. 2019 Mar 21;17(3):e2006540. doi: 10.1371/journal.pbio.2006540 (PMC6445471; doi:10.1371/journal.pbio.2006540)

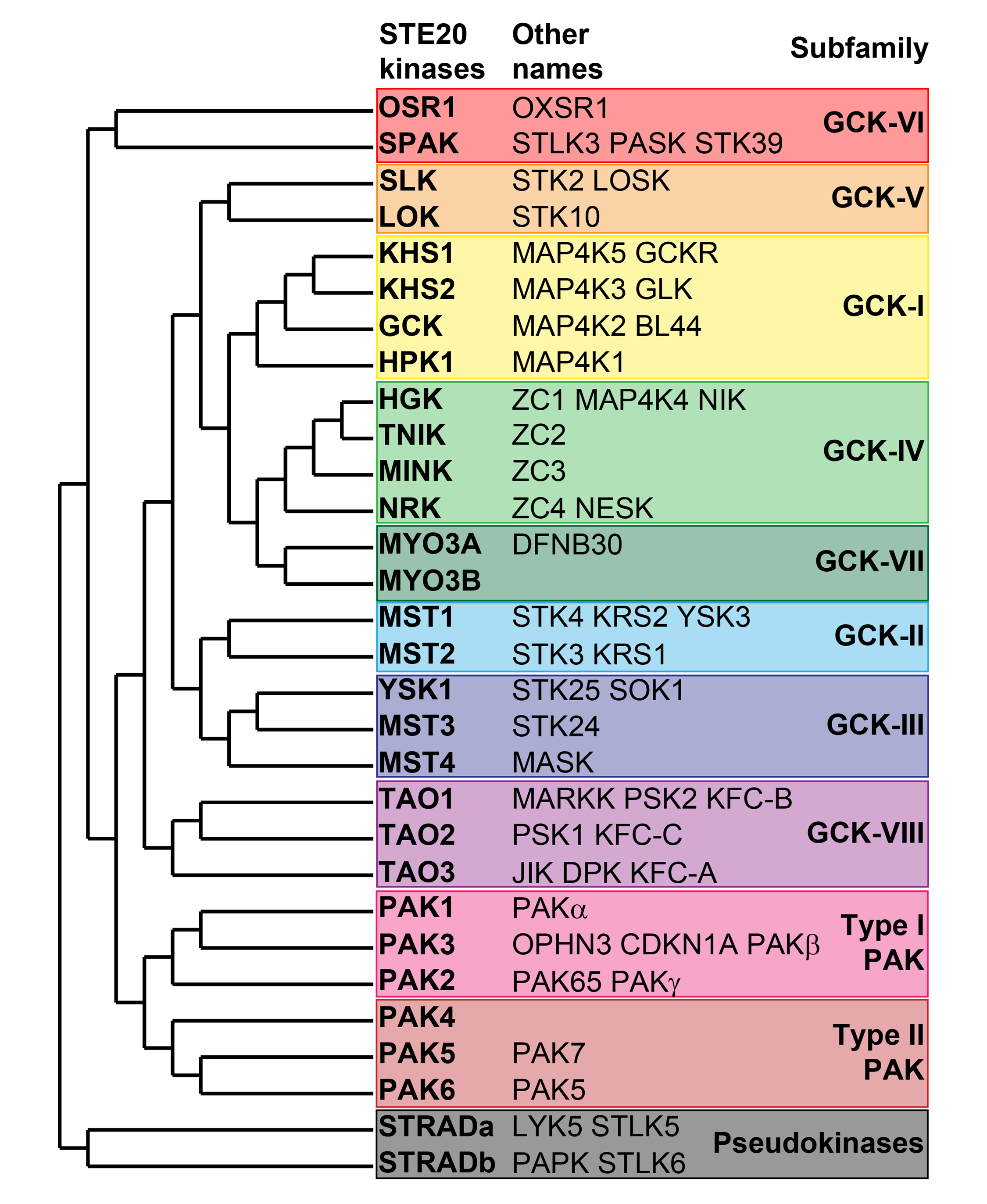

Supplement: S1 Fig — Alternative names and subfamily notation as originally proposed by Dan and colleagues [8] are shown. GCK, germinal center kinase; HGK, HPK/GCK-like kinase; HPK1, Hematopoietic progenitor kinase 1; KHS, Kinase homologous to SPS1/STE20; LOK, Lymphocyte-oriented kinase; MINK1, Misshapen-like kinase 1; MST, Mammalian sterile 20 kinase; MYO3, myosin-III; NRK, NIK-related protein kinase; OSR1, Oxidative stress-responsive 1; PAK, p21-activated kinase; SLK, STE20-like kinase; SPAK, STE20/SPS1-related proline-alanine-rich protein kinase; STRAD, STE20-related kinase adapter protein; TAO, thousand and one amino acid kinase; TNIK, Traf2 and NCK-interacting protein kinase; YSK1, Yeast Sps1/Ste20-related Kinase 1. (TIF) [file pbio.2006540.s001.TIF]

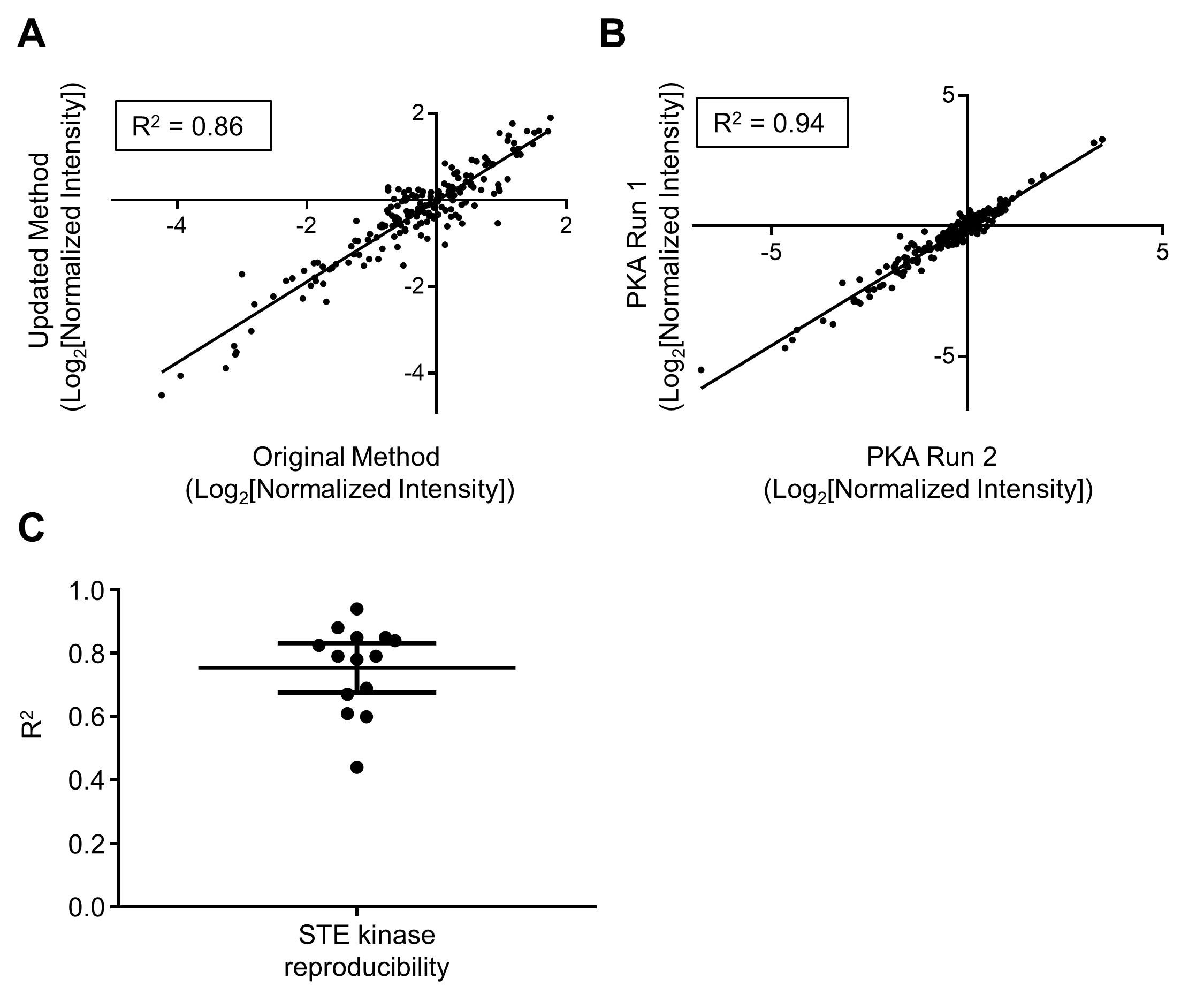

Supplement: S2 Fig — (A) Comparison of the Log2 transformed, normalized values for MST4 analyzed using the fully manual PSPA method [81] and the semiautomated method. (B) Comparison of the Log2 transformed values from two separate analyses of cyclic PKA using the semiautomated PSPA method. (C) Correlation between normalized values in two replicate PSPA assays for each STE20 kinase analyzed using the semiautomated method. Average R2 values for all replicates were 0.75 ± 0.14, with most showing R2 > 0.6. For the outlier kinase with the lowest correlation (MST3), two additional PSPA assays were performed. Error bars indicate 95% CIs. Numerical values used to generate all graphs are provided in S3 Data. MST, Mammalian sterile 20 kinase; PKA, cAMP-dependent protein kinase; PSPA, positional scanning peptide array. (TIF) [file pbio.2006540.s002.TIF]

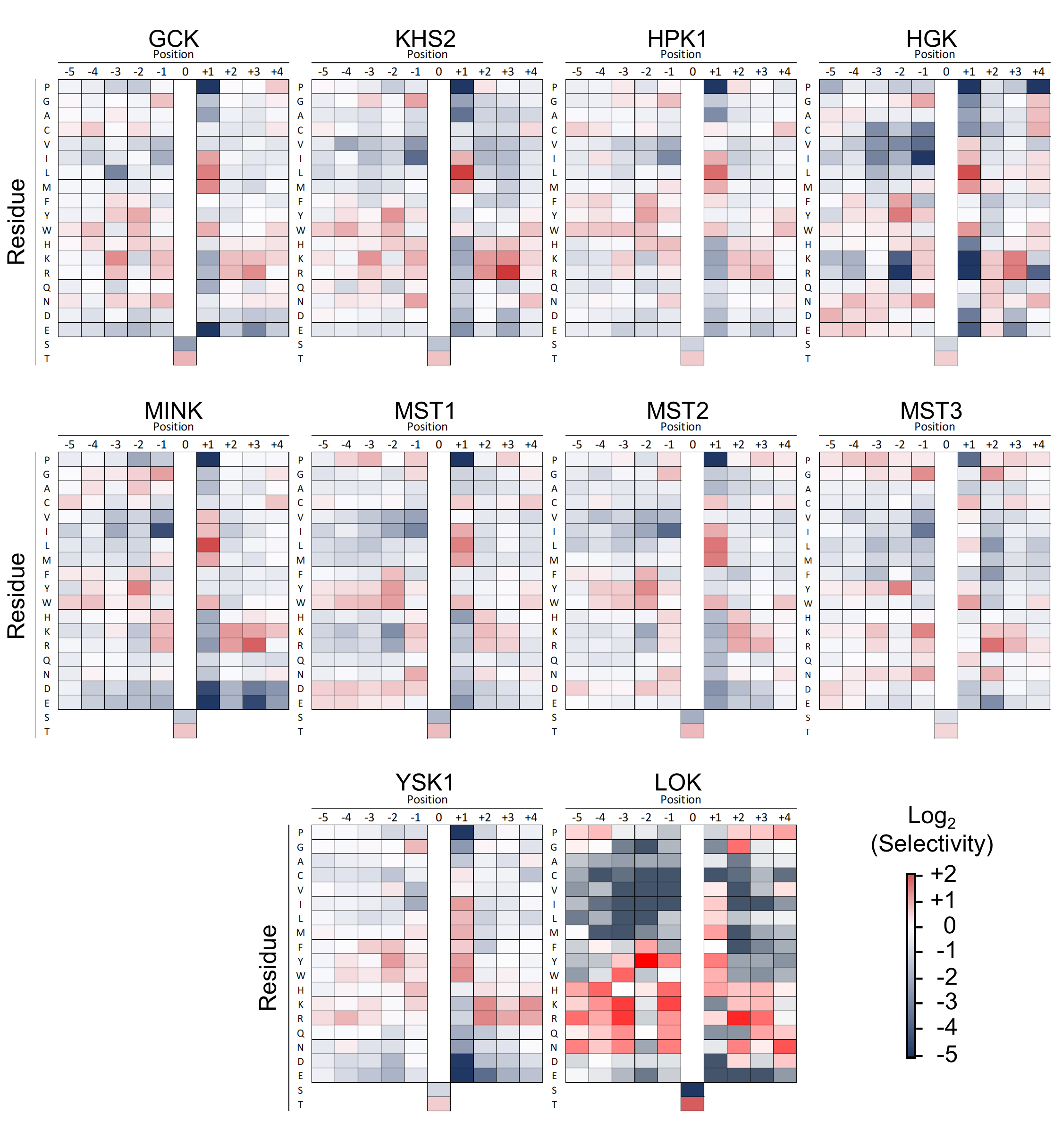

Supplement: S3 Fig — Heat maps and sequence logos were generated from PSPA data averaged from at least two experiments as in Fig 1. PSPA, positional scanning peptide array. (TIF) [file pbio.2006540.s003.TIF]

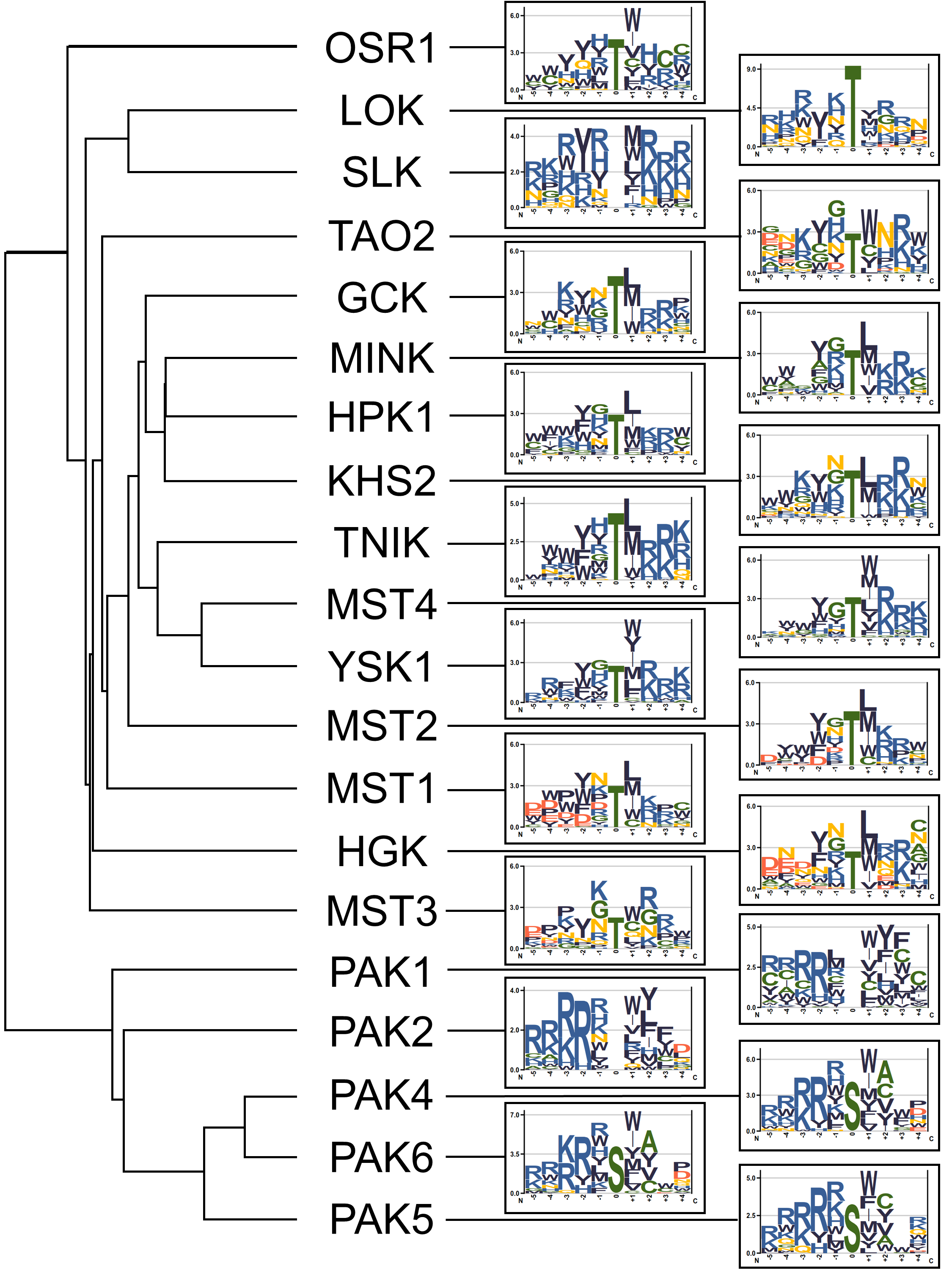

Supplement: S4 Fig — Single linkage hierarchical clustering of PSPA data was performed using Spearman rank correlation (cluster 3.0). Before clustering analysis, PSPA data were Log2 transformed (with −3 being the lowest allowed value), and Ser/Thr data were removed from all but the 0 position. Logos were generated from positive PSPA selections using enoLOGOS [44]. PSPA, positional scanning peptide array. (TIF) [file pbio.2006540.s004.TIF]

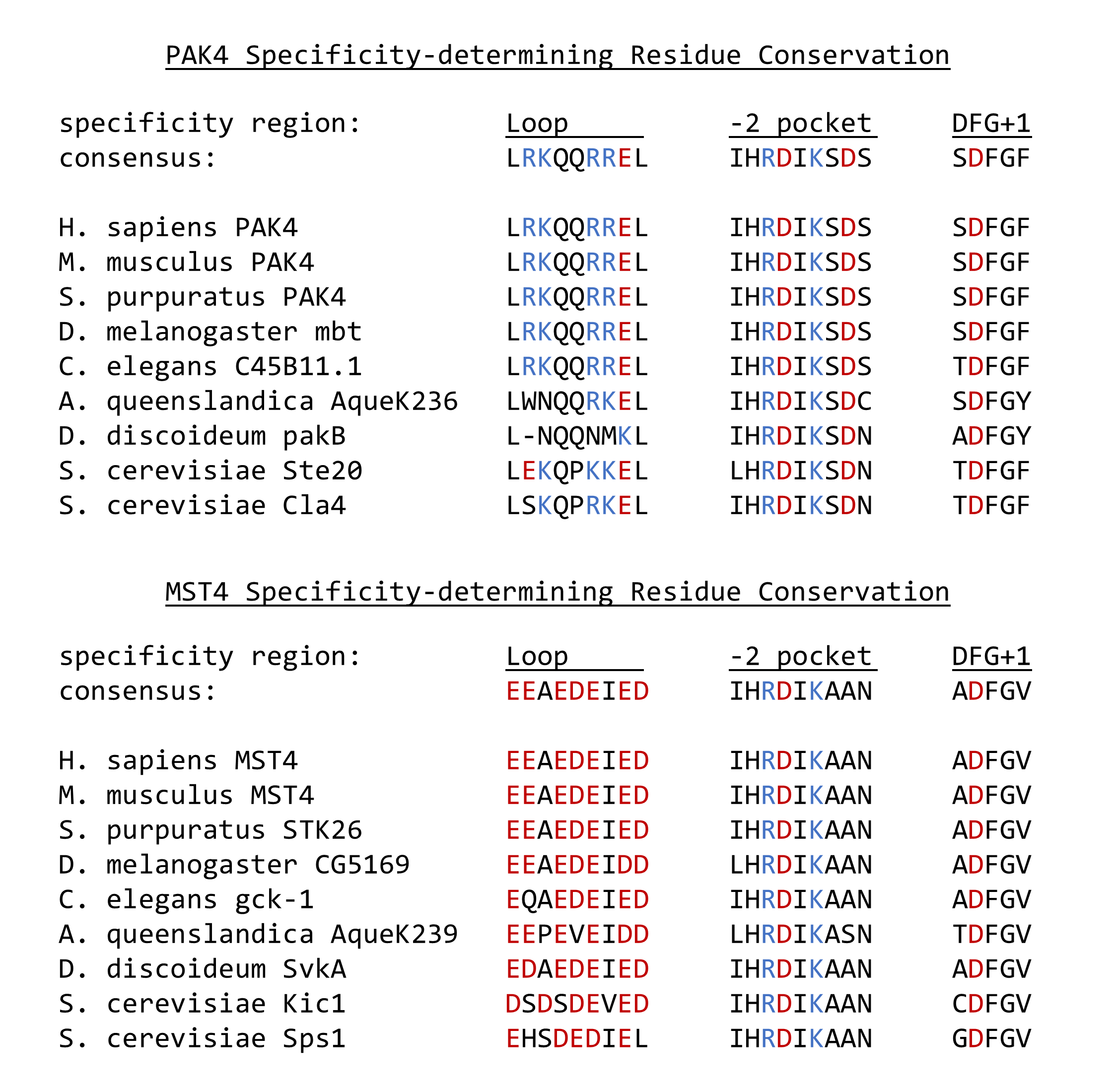

Supplement: S5 Fig — MST, Mammalian sterile 20 kinase; PAK, p21-activated kinase. (TIF) [file pbio.2006540.s005.TIF]

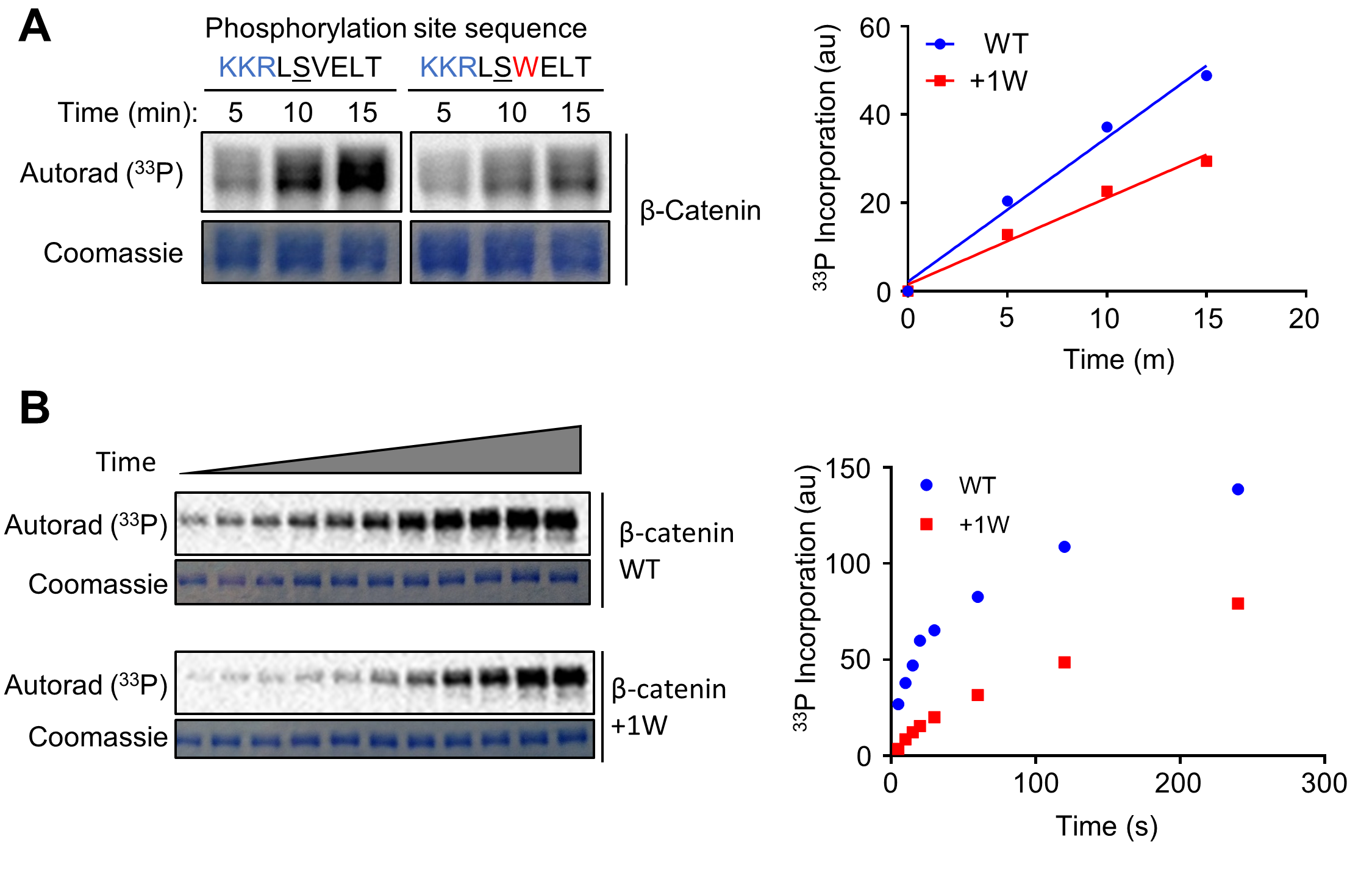

Supplement: S6 Fig — Kinase assay with full-length WT and +1W (V676W) β-catenin under (A) Michaelis–Menten and (B) single turnover conditions. Data used to generate graphs are provided in S3 Data. PAK, p21-activated kinase; WT, wild type. (TIF) [file pbio.2006540.s006.TIF]

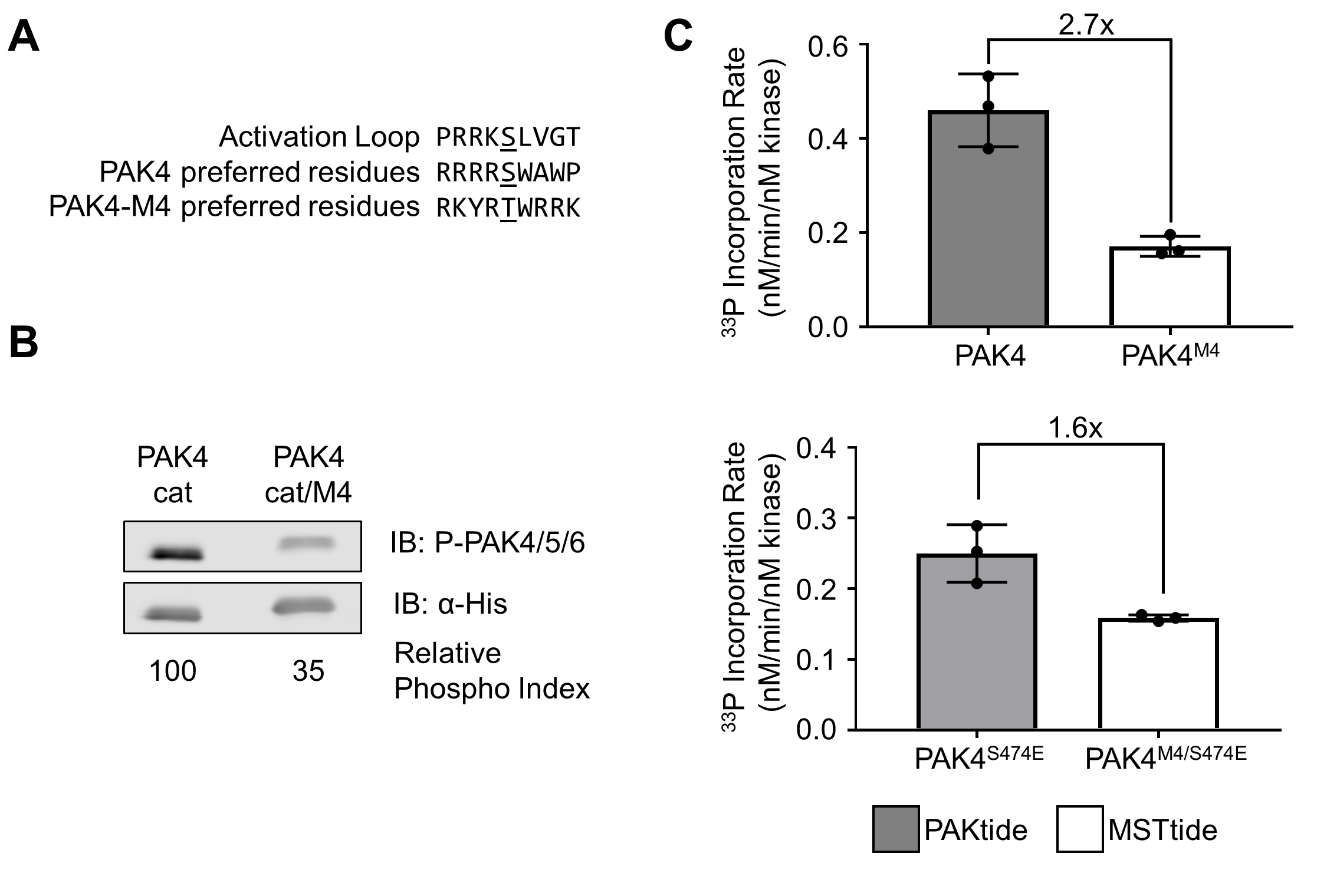

Supplement: S7 Fig — (A) The PAK4 activation loop phosphorylation site sequence conforms to the WT PAK4 recognition motif but is likely to be disfavored by PAK4M4. (B) Bacterially expressed PAK4M4 catalytic domain has reduced activation loop phosphorylation relative to its WT counterpart. (C) Peptide kinase assays on respective preferred substrates show that introduction of the activation loop phosphomimetic S474E mutation improves the activity of full-length PAK4M4 relative to WT PAK4 (n = 3, error bars represent SD). Data used to generate the graphs are provided in S3 Data. Kinases were expressed and purified from HEK293T cells. HEK, human embryonic kidney; PAK, p21-activated kinase; WT, wild type. (TIF) [file pbio.2006540.s007.tif]

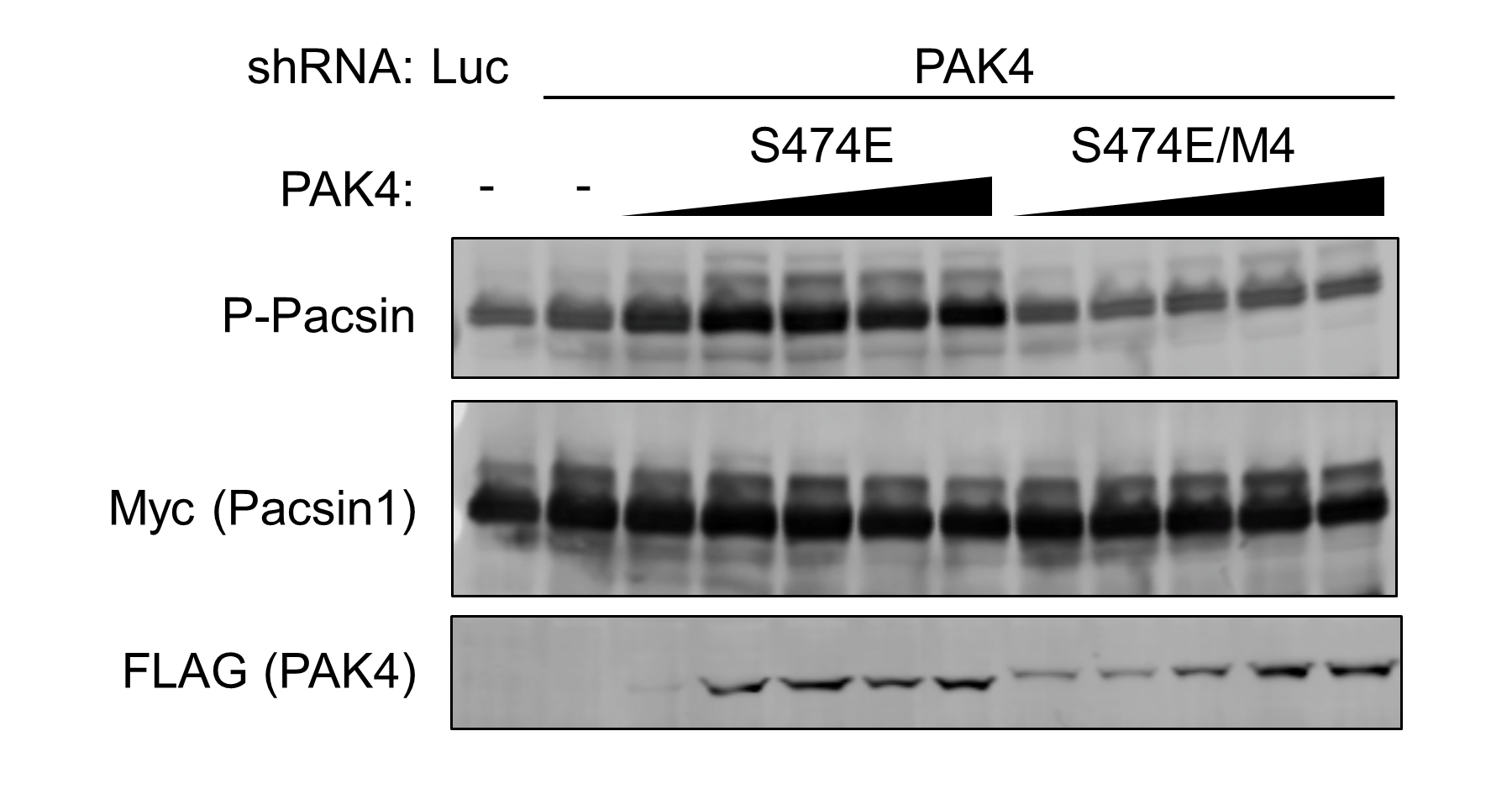

Supplement: S8 Fig — Panc1 cells expressing inducible shRNA directed to PAK4 or luciferase were co-transfected in 6-well plates with an expression plasmid for Myc–Pacsin1 and increasing amounts of plasmids expressing the indicated PAK4 mutant. Each well received 3 μg of Pacsin1 plasmid and 1 μg total of empty vector mixed with PAK4 expression vector. Quantities of PAK4 vector ranged from 0.2 μg to 1.0 μg. After incubation for 40 hours and serum starvation, cell lysates were prepared and immunoblotted with the indicated antibodies. Pacsin1, Protein kinase C and casein kinase substrate in neurons protein 1; PAK, p21-activated kinase; shRNA, short hairpin RNA. (TIF) [file pbio.2006540.s008.tif]

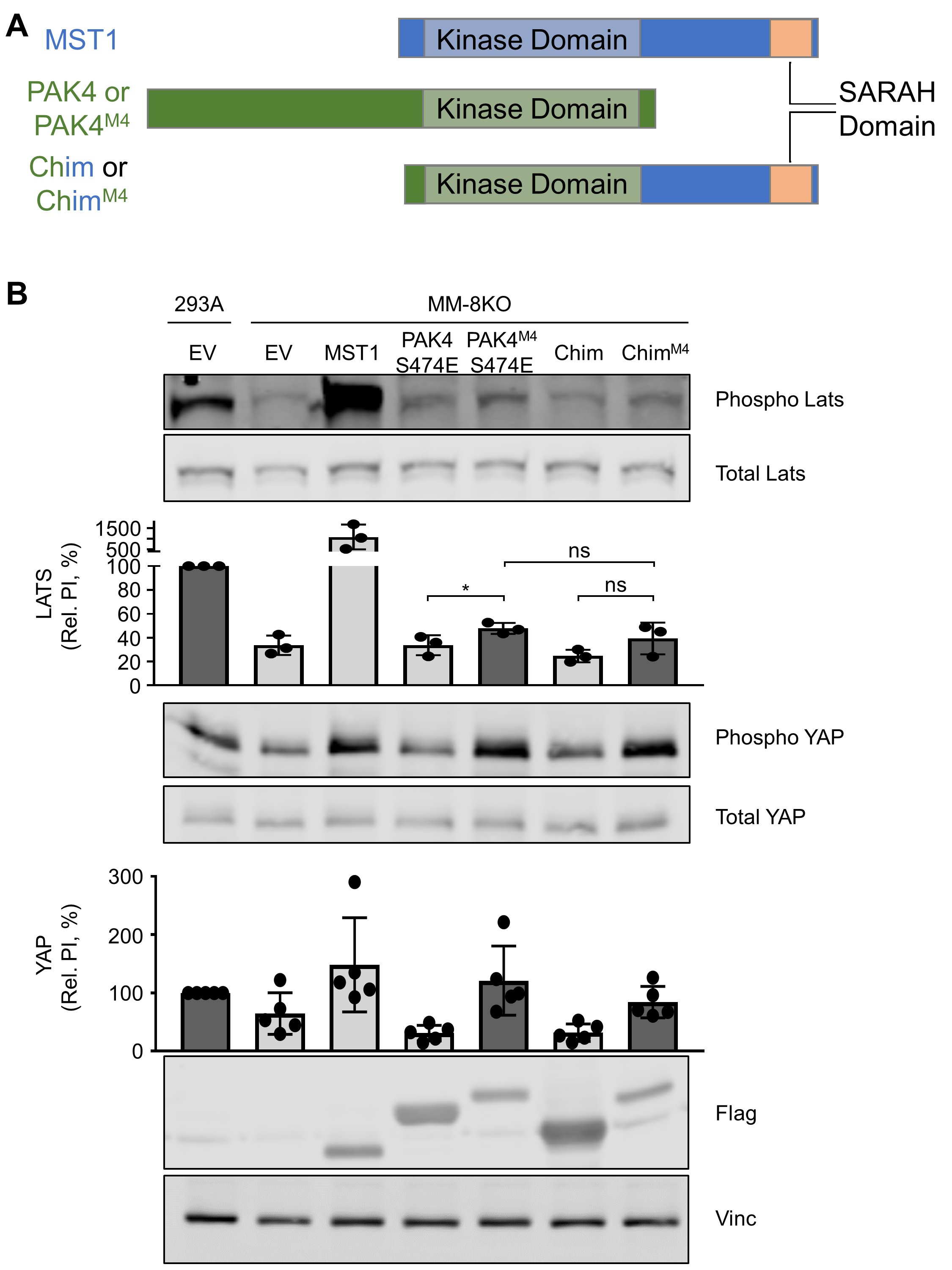

Supplement: S9 Fig — (A) Schematic representation of chimeric kinases. The MST1 kinase domain was exchanged with either PAK4S474E (Chim) or PAK4M4/S474E (ChimM4). (B) As in Fig 7A, the indicated kinases were transiently expressed in either the parental or MM-8KO 293A cells, and lysates were analyzed by immunoblotting. A representative blot from three independent experiments is shown, along with quantification of the Rel. PIs (%) of the LATS and YAP blots. Differences in LATS activation were tested using a paired t test (*p < 0.05, ns = not significantly different at p = 0.05). Error bars indicate SD. Data used to generate the graphs are provided in S3 Data. LATS, large tumor suppressor homolog; MST, Mammalian sterile 20 kinase; PAK, p21-activated kinase; Rel. PI, relative phosphorylation index; YAP, Yes-associated protein. (TIF) [file pbio.2006540.s009.tif]

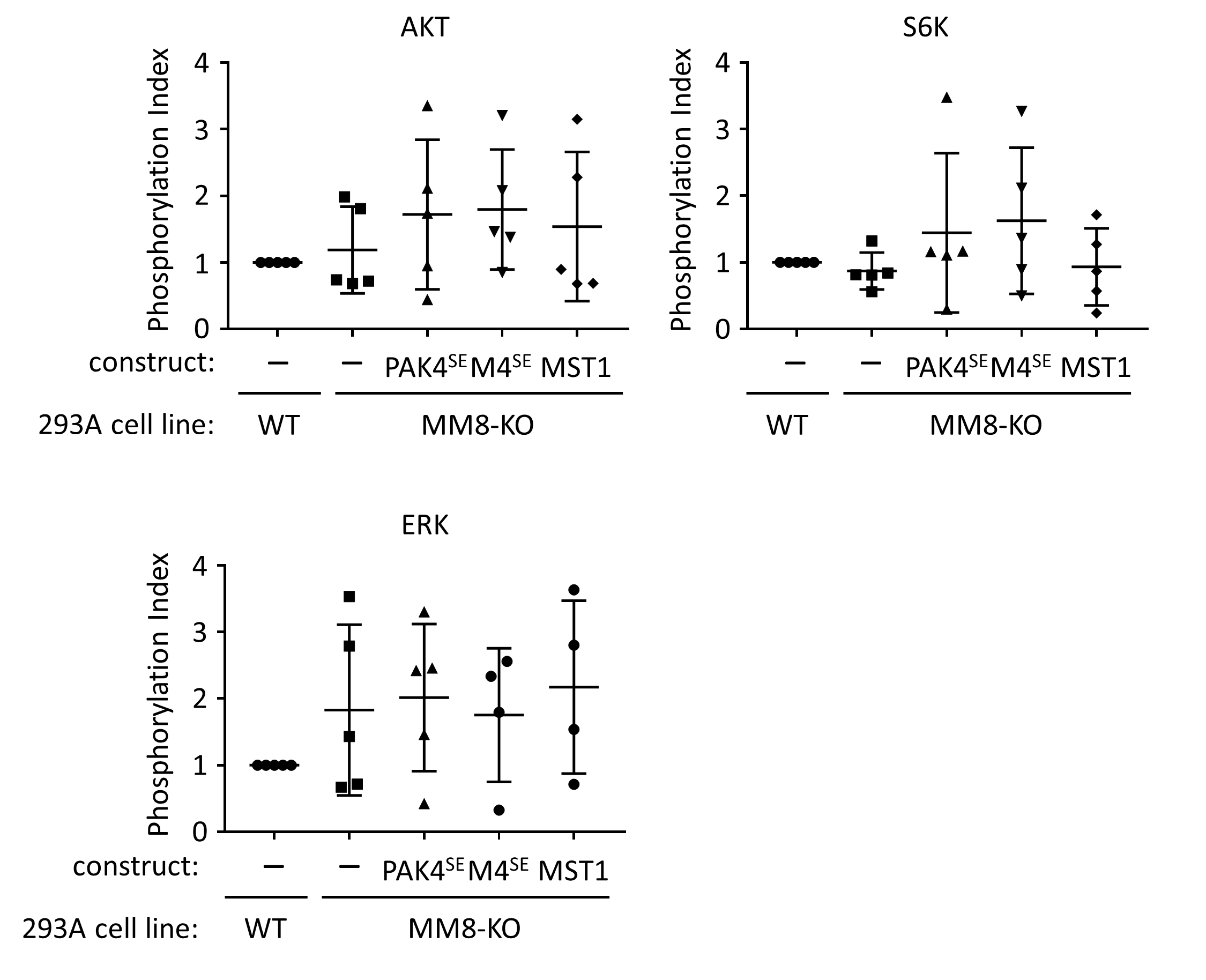

Supplement: S10 Fig — Changes in the phosphorylation states of established growth pathways in HEK293A parental and MM-8KO cells upon transfection with PAK4SE and MST1 constructs. Cells lysates were immunoblotted for total and phospho-AKT (pS473), phospho-S6K (pT389), and phospho-Erk (pT202/pY204). The phosphorylation index (phosphorylation signal/total signal) was normalized to the 293A parental value for each experiment. Error bars represent SD, and each experiment was performed at least four times. Data used to generate graphs are provided in S3 Data. Erk, Extracellular signal-regulated kinase; HEK, human embryonic kidney; MST, Mammalian sterile 20 kinase. (TIF) [file pbio.2006540.s010.TIF]
