# Supplementary material for: Comprehensive profiling of the STE20 kinase family defines features essential for selective substrate targeting and signaling output
Source: PLoS Biol. 2019 Mar 21;17(3):e2006540. doi: 10.1371/journal.pbio.2006540 (PMC6445471; doi:10.1371/journal.pbio.2006540)
Supplement: S1 Table — (PDF) [file pbio.2006540.s014.pdf]

Table S1. List of primers used used in this study. OEPCR, overlap extension PCR.

Purpose	Template(s)	Notes	Primer (5' to 3')
Add attB site and Kozak sequence	PAK4	Forward	GGGGACAAGTTTGTACAAAAAAGCAGGCTTCACCATGTTTGGGAAGAGGAAGAAGC
		Reverse	GGGGACCACTTTGTACAAGAAAGCTG GGTCTCTGGTGCGGTTCTGGCG
Add attB site and Kozak sequence	TAOK2	Forward	GGGGACAAGTTTGTACAAAAAAGCAGGCTTTAAGACCATGCCAGCTGGGGGCCGG
		Reverse	GGGGACCACTTTGTACAAGAAAGCTGGGTCTGCCTCTTGGAACAGGATCTTCTTC
Kinase dead mutation	GCK	D136N Forward	GGGAAGATCCACAGAAACATCAAGGGAGCCAAC
		D136N Reverse	GTTGGCTCCCTTGATGTTTCTGTGGATCTTCCC
Kinase dead mutation	HGK	D153N Forward	ATCATGTGATTCACCGGAATATCAAGGGCCAGAATG
		D153N Reverse	CATTCTGGCCCTTGATATTCCGGTGAATCACATGAT
Kinase dead mutation	HPK1	D137N Forward	CAGAAGAAGATACACAGGAACATCAAGGGAGCTAACATC
		D137N Reverse	GATGTTAGCTCCCTTGATGTTCCTGTGTATCTTCTTCTG
Kinase dead mutation	KHS2	D136N Forward	CACAGTAAAGGAAAAATGCACAGAAATATAAAGGGAGCTAACATTCTATTAAC
		D136N Reverse	GTTAATAGAATGTTAGCTCCCTTTATATTTCTGTGCATTTTTCCTTTACTGTG
Kinase dead mutation	MINK	D153N Forward	CAAGGTGATCCATCGAAACATCAAGGGGCAG
		D153N Reverse	CTGCCCCTTGATGTTTCGATGGATCACCTTG
Kinase dead mutation	mMST1	D149N Forward	GAGAAAAATACACCGAAATATCAAGGCGGGAAATATTTTGC
		D148N Reverse	GCAAAATATTTCCCGCCTTGATATTTCGGTGTATTTTTCTC
Kinase dead mutation	MST2	D146N Forward	CACTTTATGAGAAAAATACACAGAAATATAAAAGCTGGAAATATTCTCCTC
		D146N Reverse	GAGGAGAATATTTCCAGCTTTTATATTTCTGTGTATTTTTCTCATAAAGTG
Kinase dead mutation	MST3	D156N Forward	GGAGAAGAAAATCCACAGAAACATTAAAGCGGCCAACG
		D156N Reverse	CGTTGGCCGCTTTAATGTTTCTGTGGATTTTCTTCTCC
Kinase dead mutation	TAOK2	D151N Forward	CACAACATGATCCATAGGAATGTGAAGGCTGGAAACATC
		D151N Reverse	GATGTTTCCAGCCTTCACATTCCTATGGATCATGTTGTG
Kinase dead mutation	PAK4	D440N Forward	GTCATCCACCGGAACATCAAGAGCGAC
		D440N Reverse	GTCGCTCTTGATGTTCCGGTGGATGAC
PKC loop mutation	PKCβ	D381A/D382A/D383A Forward	GTTGTGATCCAAGCTGCTGCCGTGGAGTGCAC
		D381A/D382A/D383A Reverse	GTGCACTCCACG GCAGCAGCTTGGATCACAAC
PAK KxxN mutation	PAK4	S443A/D444A Forward	CATCCACCGGGACATCAAGGCCGCCTCGATCCTGCTGACCCATG
		S443A/D444A Reverse	CATGGGTCAGCAGGATCGAGGCGGCCTTGATGTCCCGGTGGATG
PAK S445N activating mutation	PAK4 S443A/D444A	S445N (on KxxN background) forward	CCGGGACATCAAGGCCGCCAACATCCTGCTGACCCATGATG
		S445N (on KxxN background) reverse	CATCATGGGTCAGCAGGATGTTGGCGGCCTTGATGTCCCGG
	PAK4	R359E/L362D Forward	GACCTGCGCAAGCAGCAGGAGCGCGAGGACCTCTTCAACGAGGTGGTAATC

PAK Loop step 1 mutation		R359E/L362D Reverse	GATTACCACCTCGTTGAAGAGGTCCTCGCGCTCCTGCTGCTTGCGCAGGTC
PAK Loop step 2 mutation	PAK4 R359E/ L362D	R355E/K356E/Q357A/Q358ED/R360I Forward	CGTCAAGAAGATGGACCTGGAGGAAGCAGAAGATGAGCGCGAGGACCTCTTC
		R355E/K356E/Q357A/Q358ED/R360I Reverse	GAAGAGGTCCTCGCGCTCATCTTCTGCTTCCTCCAGGTCCATCTTCTTGACG
PAK DFG+1 mutation	PAK4	F461V Forward	TGTCAGACTTTGGGGTCTGCGCCCAGGTG
		F461V Reverse	CACCTGGGCGCAGACCCCAAAGTCTGACA
MST4 K<u>xx</u>N mutation	MST4	A147S/A148D Forward	GAAAATTCACCGAGACATAAAATCTGACAATGTCTTGCTCTCAG
		A147S/A148D Reverse	CTGAGAGCAAGACATTGTCAGATTTTATGTCTCGGTGAATTTTC
MST4 Loop step 1 mutation	MST4	E63K/D66L Forward	CTTGAGGAAGCCGAAGATAAAATAGAACTCATTCAGCAAGAAATAACT
		E63K/D66L Reverse	CAGTTATTTCTTGCTGAATGAGTTCTATTTTATCTTCGGCTTCCTCAA
MST4 Loop step 2 mutation	MST4 E63K/D66L	E58R/E59K/A60Q/E61Q/D62X/I64R Forward	GCTATTAAAATCATAGACCTTCGCAAGCAGCAGAAAATAGAACTCATTCAGC
		E58R/E59K/A60Q/E61Q/D62X/I64R Reverse	GCTGAATGAGTTCTATTTTCTGCTGCTTGCGAAGGTCTATGATTTTAATAGC
MST4 DFG+1 mutation	MST4	V165F Forward	GCTGATTTTGGATTTGCTGGTCAGCTG
		V165F Reverse	AGCTGACCAGCAAATCCAAAATCAGC
Create full length PAK4-M4 by OEPCR	PAK4 FL	Forward of PAK4 FL (with attB and Kozak)	GGGGACAAGTTTGTACAAAAAAGCAGGCTTCACCATGTTTGGGAAGAGGAAGAAGCGGG
		Reverse overlap	GAGCGGGGGCCAGGGGGCCCAGG AACAGCAGGGGCG
	PAK4-cat	Forward overlap	CGCCCCTGCTGTTCCTGGGCCCCCTGGCCCCCGCTC
		Reverse (with attB)	GGGGACCACTTTGTACAAGAAAGCTGGGTCTCTGGTGCGGTTCTGG CGCATG
PAK phosphomimetic mutation	PAK4 and PAK4-M4	S474E Forward	CCCCGAAGGAAGGAGCTGGTCGGCACGC
		S474E Reverse	GCGTGCCGACCAGCTCCTTCCTTCGGGG
PAK4 pcDNA-Flag subcloning	PAK4^{S474E} and PAK4-M4^{S474E}	Forward	ATGACGATAAAGCAAGGGATCCGTTTGGGAAGAGGAAGAAGC
		Reverse	CGGCCGTTACTAGTGGATCCTCACTATCTGGTGCGGTTCTG
shRNA resistant PAK4	PAK4	Forward	GCAGCAGAGGCGCGAGCTACTATTTAACGAGGTGGTAATCATGAG
		Reverse	CTCATGATTACCACCTCGTTAAATAGTAGCTCGCGCCTCTGCTGC
YSK1 E293*	YSK1-cat	Forward	CGCTATAAGCGCTGGAAGTCATAGGGGCATGGC
		Reverse	GCCATGCCCCTATGACTTCCAGCGCTTATAGCG
Create PAK4-MST1 and PAK4^{M4}-MST1 chimeras by OEPCR	PAK4 cat or PAK4^{M4}cat	Forward of PAK4cat (with attB and Kozak)	GGGGACAAGTTTGTACAAAAAAGCAGGCTTCACCATGGCCCGTGCCCGCCAGGAAAATGG
		Reverse overlap	CTTCCTGGCGCTTCAGTTTCACATCTCTGGTGCGGTTCTGGCGCATG
	MST1	Forward overlap	CATGCGCCAGAACCGCACCAGAGATGTGAAACTGAAGCGCCAGGAAG
		Reverse (with attB)	GGGGACCACTTTGTACAAGAAAGCTGGGTCGAAGTTCTGTTGCCTCCTCTTCTTG
Create β-catenin minor PAK4	β-catenin	S552A Forward	GTTCCACCCATGGCGGTGCGCCGTTGG

sites mutant		S552A Reverse	CCAACGGCGCACCGCCATGGGTGGAAC
		T556A Forward	CCTCCATGGGTGGAGCGCAGCAGCAG
		T556A Reverse	CTGCTGCTGCGCTCCACCCATGGAGG
Create +1W β-catenin	β-catenin S552A/T556A	V676W Forward	CAAGAAGCGGCTTTCATGGGAGCTGACCAGTTC
		V676W Reverse	GAACTGGTCAGCTCCCATGAAAGCCGCTTCTTG
Create phospho-resistant β-catenin	β-catenin S552A/T556A	S675A Forward	GATTACAAGAAGCGGCTTGCAGTCGAGCTGACCAGTTC
		S675A Reverse	GAACTGGTCAGCTCGACTGCAAGCCGCTTCTTGTAATC
β-catenin MST motif mutation step 1	β-catenin S552A/T556A	K672G/R673Y/L674N/S675T Forward	GCCACAGGATTACAAGGGATATAATACAGTCGAGCTGACCAG
		K672G/R673Y/L674N/S675T Reverse	CTGGTCAGCTCGACTGTATTATATCCCTTGTAATCCTGTGGC
β-catenin MST motif mutation step 2	β-catenin S552A/T556A/ K672G/R673Y/L674N/S675T	E677R/L678R/T679K/S680K Forward	GGATATAATACAGTCCGGCGGAAAAAATCCCTCTTCAGGACAG
		E677R/L678R/T679K/S680K Reverse	CTGTCCTGAAGAGGGATTTTTTCCGCCGGACTGTATTATATCC
β-catenin MST phospho-resistant control	β-catenin MST	T675A Forward	CAAGGGATATAATGCAGTCCGGCGGAAAAAATC
		T675A Reverse	GATTTTTTCCGCCGGACTGCATTATATCCCTTG
